# DeMixNB: deconvolution of sparse-count RNA sequencing data for tumor cells using embedded negative binomial distributions

**DOI:** 10.1186/s13059-026-04234-4

**Published:** 2026-08-10

**Authors:** Matthew D. Montierth, Hao Yan, Liyang Xie, Kinga Nemeth, Xiaoxi Pan, Ruonan Li, Caner Ercan, Peng Yang, Ansam Sinjab, Tieling Zhou, Fuduan Peng, Manisha Singh, Linghua Wang, Scott Kopetz, Humam Kadara, Yinyin Yuan, George A. Calin, Wenyi Wang

**Affiliations:** 1 Department of Bioinformatics and Computational Biology, The University of Texas MD Anderson Cancer Center, Houston, TX 77030 USA; 2 Graduate Program in Quantitative Computational Biology, Baylor College of Medicine, Houston, TX 77030 USA; 3 Department of Biostatistics and Data Science, The University of Texas Health Science Center at Houston, Houston, TX 77030 USA; 4 Department of Statistics and Data Sciences, The University of Texas at Austin, Austin, TX 78712 USA; 5 Department of Translational Molecular Pathology, The University of Texas MD Anderson Cancer Center, Houston, TX 77030 USA; 6 Institute of Data Science in Oncology, The University of Texas MD Anderson Cancer Center, Houston, TX 77030 USA; 7 Department of Gastrointestinal Medical Oncology, The University of Texas MD Anderson Cancer Center, Houston, TX 77030 USA; 8 Department of Genomic Medicine, The University of Texas MD Anderson Cancer Center, Houston, TX 77030 USA; 9 Department of Cancer Biology, The University of Texas MD Anderson Cancer Center, Houston, TX 77030 USA; 10 The Center for RNA Interference and Non-Coding RNAs, The University of Texas MD Anderson Cancer Center, Houston, TX 77030 USA

**Keywords:** Tumor-immune contact, Prognosis biomarker, Total miRNA expression, Total mRNA expression

## Abstract

Estimating tumor-specific transcript proportions from mixed bulk samples has potential to inform novel biology. However, estimation accuracy using existing methods in sparse-count data such as microRNA-seq and spatial transcriptomics has yet to be established. We generate a mixed small RNA benchmark dataset to demonstrate analytical challenges. To resolve them, we develop DeMixNB, a semi-reference-based deconvolution model assuming a sum of negative binomial distributions. Applications to miRNA-seq from 885 patients with breast cancer and 4,709 spatial spots from lung cancer generates clinical and mechanistic insights into tumor cell plasticity. This supports the important utility of DeMixNB to investigate cancer RNomes.

**Supplementary Information:**

The online version contains supplementary material available at https://doi.org/10.1186/s13059-026-04234-4.

## Background

Cancer accounts for nearly one in six deaths globally and is a leading cause of mortality across both developed and developing nations [1]. Despite decades of research and improvements in therapies, treatment outcomes vary, reflecting not only differences in access and care but also profound biological variation between tumors [2]. Cancer’s complexity partly stems from the presence of intratumor heterogeneity and the dynamic nature of tumor cell plasticity [3, 4]. Variability within a tumor arises from the complex cellular composition in the local tumor microenvironment (TME), impacting progression, spread and therapeutic resistance [5, 6]. Adding to this cellular complexity, the plasticity of malignant cells, which is the ability of cancer cells to transit between distinct stages of differentiation and a state of ‘stemness’, fosters the tumor diversity [7, 8]. Such plasticity-driven variations significantly influence the total amount of transcripts originating from tumor cells [9, 10]. Recent studies [11] showed that tumor cells exhibit great transcriptional activity and variation, discordant with variations presented in surrounding non-tumor cells. Therefore, accurately quantifying tumor-cell specific transcript proportion of tissues offers direct insights into tumor heterogeneity and is becoming a unique and desirable feature to obtain for cancer studies [12].

One common computational strategy to perform the aforementioned task is deconvolution, a mathematical technique that can separate mixed signals from tissues, enabling the estimation of cell-type proportions, for either cell counts or cell transcripts, and cell specific transcript proportion to reveal tumor heterogeneity [13]. The most widely used deconvolution framework is reference-based deconvolution techniques, which requires finding marker genes with stably expressed profiles for all cell types of interest. It is therefore not suitable to capture the yet-to-be-measured transcriptional plasticity in tumor cells. Reference-free methods [14–19], on the contrary, make minimal assumptions on the cell-type specific gene expression profiles, but consequently cannot achieve as good estimation performances. Semi-reference‐based deconvolution [20–28] provides a middle ground, requiring reference data from cell types that are relatively stably expressed while offering flexibility on the tumor-cell gene expression. This setup makes semi-reference-based best suited for estimating tumor-specific transcript proportions.

Existing semi-reference-based methods like DeMix [20]/DeMixT [21] and ISOpure [22] use expression profiles of adjacent normal tissues as the partial reference. Other methods, such as PREDE [25] and BayICE [28], rely on Non-negative Matrix Factorization (NMF) and Markov chain Monte Carlo procedure through Gibbs sampling, using pre-selected cell-type marker genes and a predefined number of optimal cell types to perform deconvolution. By integrating partial reference data, semi-reference methods can address the issues of instability of deconvolution results, incomplete cellular knowledge and technical variability. While these methods provide a versatile and reliable solution for dissecting the TME’s mixed cellular compositions [13], they were primarily developed for high read count data like bulk RNA sequencing (RNA-seq) data. This is a major limitation of the log-normal distribution assumption in DeMixT, preventing it from applying to the deconvolution of several important RNomes, such as microRNA sequencing [13, 29] (miRNA-seq) or spot-level spatial transcriptomic (ST) data [30–32].

MiRNAs and other non-coding RNAs are extensively linked to biology and disease pathogenesis [33], although no single-cell sequencing technology has been established for miRNA. Spatial transcriptomics from popular technologies such as 10X Genomics Visium faces two major analytical challenges: sparsity from low capture efficiency and mixed signals from each spot capturing 5–50 cells of multiple cell types. Reference-based deconvolution methods have been developed recently for both bulk miRNA-seq (DeconmiR [34]), and ST (e.g., RCTD [35], CARD [36]) data. Still, no semi-reference-based methods have been developed to study tumor-cell transcript proportions in these data types.

**Fig. 1:**
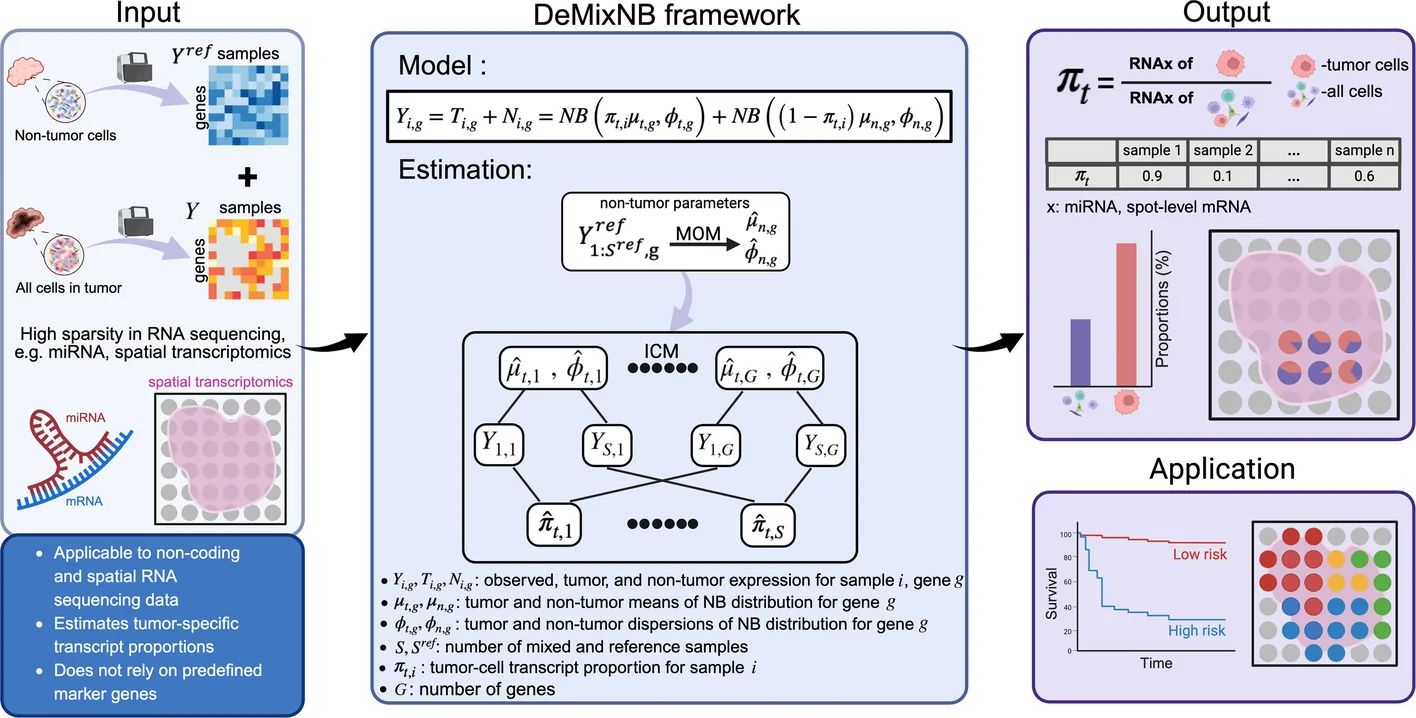
Overview of DeMixNB. DeMixNB takes gene expression profiles from mixed samples and corresponding reference samples as input (sample-by-gene for bulk sequencing data or spot-by-gene for spatial transcriptomic data). DeMixNB estimates tumor-specific transcript proportions without the need for external reference or cell type marker gene list. Observed gene expression ${Y}_{i,g}$ is modeled as the sum of reference component ${N}_{i,g}$ and tumor component ${T}_{i,g}$, with each following a negative binomial distribution, respectively. Tumor-transcript proportion ${\pi}_{t,i}$ is estimated simultaneously with the mean ${\mu}_{t,g}$ and overdispersion parameter ${\phi}_{t,g}$. For each mixed sample, DeMixNB estimates ${\pi}_{t}$, which is the tumor transcript proportion of the RNA species being studied. In spatial transcriptomics, this is estimated for each spot, which can be used to refine profiling of spatial patterns in tumor tissues. For sparse non-coding RNAs like miRNAs, the ${\pi}_{t}$-miR proportion can be used as a biological feature in downstream analyses. Similar application is also feasible for the spot-level ${\pi}_{t}$. For simplicity, we use π to represent ${\pi}_{t}$ henceforth in this paper. MOM: Method of moments. ICM: iterated conditional modes

To fill this gap, we have developed DeMixNB that models the tumor and non-tumor cell compartments as individual negative binomial (NB) distributions for low read counts with overdispersion and takes the reference profiles from the non-tumor cell compartment only as input (Fig. 1). Using our specially designed cell-line mixing miRNA experiment as benchmark for low read count data, we found DeMixNB to significantly outperform existing methods in estimating tumor-cell transcript proportions. We further applied DeMixNB to two datasets obtained from published studies [37–39] to demonstrate that for both miRNA-seq and ST Visium data, tumor-cell transcript proportions are distinct from tumor-cell count proportions, i.e., tumor purity. The estimated tumor-cell transcript proportions have the potential to inform prognosis-associated transcriptional activities in tumor cells, as well as features of TME that may influence these activities. The DeMixNB function is freely available within the Bioconductor [40] R package DeMixT 2.0.

## Results

### DeMixNB overview

As we aimed to estimate tumor-cell transcript proportions accurately in at least two types of RNomes: miRNA-seq and spatial transcriptomics, we first evaluated the read count sparsity and variation in both data types. The miRNA-seq data from TCGA breast cancer presents significantly lower read counts than the mRNA-seq data of the same samples (Methods, median of the mean normalized expression levels is 1.5 and 1,017 for miRNA-seq and mRNA-seq, respectively, Additional file 1: Fig. S1), with the variance of the expression levels significantly larger than the mean while following closely negative binomial distributions (Additional file 1: Fig. S2). The spatial transcriptomic data from a lung cancer patient presents lower read counts than miRNA-seq (Methods) while presenting a similar degree of overdispersion to negative binomial distributions (Additional file 1: Fig. S2). Existing semi-reference-based deconvolution methods either assume normal or log-normal distributions, or not account for variance across samples, hence not suited for the tumor-cell transcription proportion estimation in these two data types. Previously, reference-based deconvolution methods for spatial transcriptomics have successfully addressed the overdispersion inherent in such data through negative binomial (NB) modeling [41, 42]. Therefore, we propose DeMixNB, a semi-reference-based deconvolution method. DeMixNB combines the established iterated estimation procedure of DeMixT with a likelihood formula for a mixture of negative binomial distributions so that the desired transcript proportions can be directly obtained through maximum likelihood estimation (Methods).

In DeMixNB, the observed expression for each gene *g* and sample *i*, ${Y}_{ig}$, is assumed to be the sum of expressions in tumor cells, ${T}_{ig}$, and expressions in non-tumor cells, ${N}_{ig}$. Both ${T}_{ig}$ and ${N}_{ig}$ are assumed to follow negative binomial distributions specified by the mean and overdispersion parameters ${\mu}_{tg}, {\phi}_{tg},$ and ${\mu}_{ng},{\phi}_{ng}$, respectively. The tumor-specific transcript proportion parameter, ${\pi}_{i}$, with *t *omitted in the notation for simplicity, ensures the mean of mixed expressions $E\left({Y}_{ig}\right) = {\pi}_{i}{\mu}_{tg }+\left(1-{\pi}_{i}\right){\mu}_{ng}$, whose estimate approximates the transcript proportion $\frac{{\sum}_{g}{T}_{ig}}{{\sum}_{g}{Y}_{ig}}$ (see further in Methods). Given an input expression matrix representing mixtures of tumor and non-tumor cells and a reference expression matrix representing only non-tumor cells, DeMixNB first estimates parameters for the non-tumor component ${\widehat{\mu }}_{ng}$ and ${\widehat{\phi }}_{ng}$, then performs the iterative conditional modes (ICM) [43] algorithm to efficiently maximize the exact likelihood of observing the target mixed samples: iteratively estimating the sample-wise parameters—the mixing proportions of tumor samples, and then the gene-wise parameters—the distribution parameters of individual gene expressions. The ICM approach is used to address an intrinsic weak tractability of all model parameters in the model’s full likelihood. Our key assumption is that the proportions only affect the mean of the distributions of expressions, while the dispersion parameters remain independent of proportions, in order to achieve computational tractability (Methods). Similar to DeMixT, gene selection for a goodness of fit to negative binomial is further implemented to improve the model performance (Methods).

**Fig. 2:**
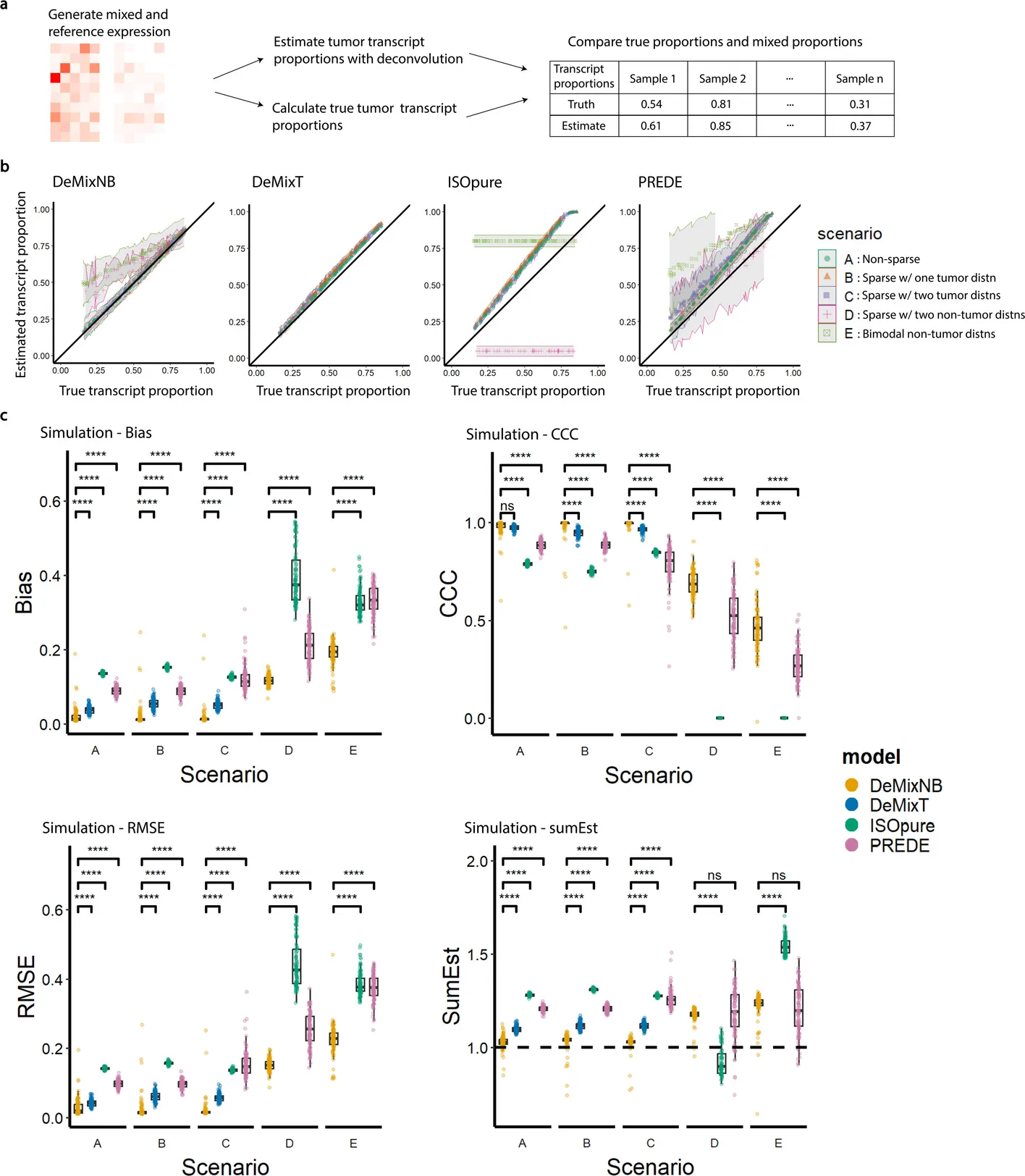
Simulation study and results (Additional file 1: Table S1). **a** An overview of the simulation process. **b** Scatter plots with confidence ribbons (mean ± standard error) for the estimated transcript proportions (y-axis) by each deconvolution method and the true transcript proportion (x-axis) calculated from the generated data. Simulation settings are marked with different colors. DeMixT did not produce results for scenarios D,E. See Methods for more details. **c** Boxplots comparing the average Bias, CCC, RMSE, and sum of estimated proportions (sumEst) over all 100 mixed samples in each of the 100 simulated datasets for DeMixNB, DeMixT, ISOpure, and PREDE, across all scenarios. For Bias and RMSE, the smaller the better. For CCC, the closer to 1, the better. The sumEst metric calculates the sum of the estimated tumor proportion using non-tumor reference and the estimated non-tumor proportions using tumor reference. The closer it is to 1, the more consistent is the estimation. All comparisons are further supported by pairwise t-tests with Benjamini-Hochberg (BH)-adjusted *p* values (significance level denoted by: *, *p* < 0.05; **, *p* < 0.01; ***, *p* < 0.001; ****, *p* < 0.0001)

*Simulation study*. To verify the estimation accuracy of DeMixNB, we considered five simulation scenarios which differ by level of sparsity and gene expression distributions: A. non-sparse count data, B. sparse count data with a single tumor-specific distribution per gene, C. sparse count data with two tumor-specific distributions across some genes representing tumor cell subtypes across samples, D. sparse count data with two non-tumor distributions across genes representing multiple non-tumor cell types, E. bimodal distributions in the reference data for a subset of genes, showcasing the benefit of gene selection (Methods). We benchmarked method performances of DeMixNB against DeMixT [21], PREDE [25] and ISOpure [22, 44], three established semi-reference-based deconvolution methods, by comparing the estimated proportions produced by each model with the true tumor-cell transcript proportions, which can be calculated as $\frac{{\sum}_{g}{T}_{ig}}{{\sum}_{g}{Y}_{ig}}$ (Fig. 2a). Our evaluation metrics include the absolute bias, concordance correlation coefficient (CCC), the root mean square error (RMSE), and the consistency metric (sumEst) that sums up the proportion estimates using the non-tumor and tumor component as reference, respectively (Methods).

Across all five scenarios, DeMixNB displayed consistently better performance in specifically estimating transcript proportions with significantly higher CCC, lower Bias and RMSE, and better consistency as measured by sumEst (Additional file 1: Table S1, Fig. 2b, c). Both DeMixNB and DeMixT performed well with non-sparse data while in this case DeMixT showed a computational speed advantage (18× faster) by assuming a continuous log-normal distribution. When applied to the sparse data scenarios, DeMixT, ISOpure, and PREDE tended to overestimate the tumor transcript proportions and were outperformed by DeMixNB. In scenario C with two different tumor-cell distributions, the most notable drop in performance was observed for PREDE as its estimation became unstable across repeated runs. Across scenarios A-C, the CCC value for DeMixNB is consistently above 0.975, representing high accuracy in estimating transcript proportions. In scenario D, the reference profile for non-tumor cells became a mixture of two negative binomial distributions, which resulted in significantly worse performance metrics for all models. DeMixNB, with a mean CCC value of 0.686 (mean Spearman correlation = 0.939), still outperformed all methods. Scenario E was designed to push the breaking point of all models, as the NB assumption is explicitly violated in the reference profiles of 200 genes. In this case, gene selection to remove those presenting a poor NB fit, i.e., with a quantile–quantile (QQ) correlation < 0.9 (~ 100 genes removed, Methods), allowed DeMixNB to achieve the best mean CCC of 0.468, better than the mean CCC of 0.271 by PREDE. The DeMixNB results are substantially better in terms of correlation between the estimates and the true proportions, with a mean Spearman correlation coefficient of 0.804 as compared to PREDE’s 0.411. This suggests in the worst-case scenario E, the order of the estimated mixing proportion can still provide a useful ranking of transcript proportions, with an estimation bias. Overall, the simulated data generated by negative binomial distributions posed challenges to all methods except DeMixNB. Such results are expected since the normality assumption for DeMixT and PREDE is explicitly violated. ISOpure assumes tumor samples belong to the same cancer subtype and models the non-tumor component without a variance parameter. These assumptions may be the cause of weak performances by ISOpure.

### DeMixNB accurately deconvolves miRNA-seq in a specially-designed mixed cell line benchmarking study

**Fig. 3:**
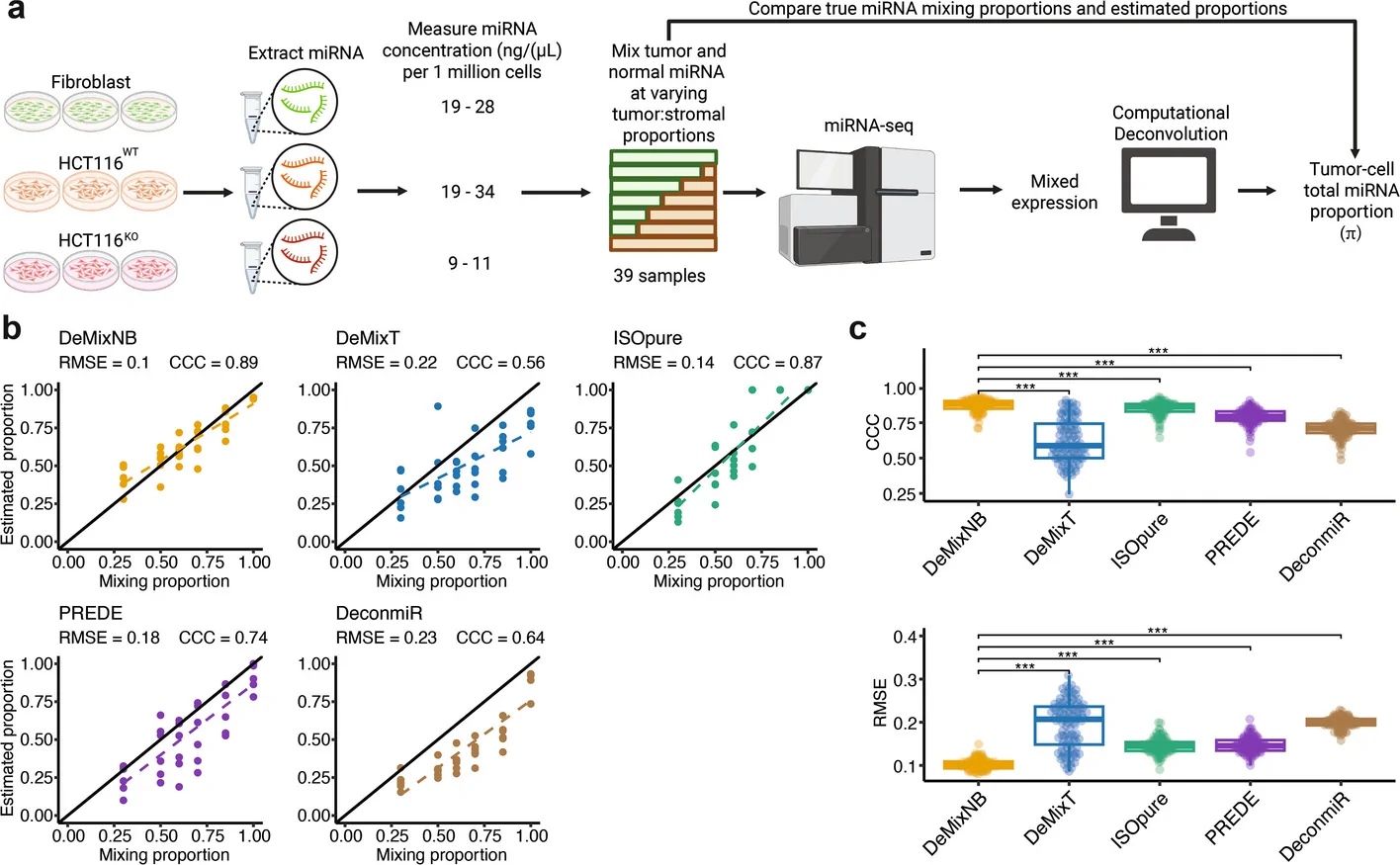
Benchmarking study design and results. **a** Graphical depiction of how the benchmarking dataset was generated (Methods). **b** Scatter plots showing the relationship between tumor-specific total miRNA transcript proportion (x-axis) and the corresponding estimated proportion (y-axis) for five methods: DeMixNB, DeMixT, ISOpure, PREDE, and DeconmiR. Each plot includes a diagonal reference line (slope = 1) and a dotted line indicating linear regression fit. RMSE and CCC values are displayed on each plot. **c** Boxplots showing bootstrapped distributions of CCC and RMSE for DeMixNB, DeMixT, ISOpure, PREDE, and DeconmiR. Pairwise Wilcoxon rank-sum test *p*-values (BH-adjusted) are indicated. For all plots, significance levels are denoted by: *, *p* < 0.05; **, *p* < 0.01; ***, *p* < 0.001

To evaluate DeMixNB's performance with sparse expression count matrices typical of bulk miRNA sequencing (miRNA-seq, Additional file 1: Figs. S1-2), we developed a benchmark dataset with precisely known cellular compositions (Fig. 3a), a strategy that has been proven to be useful in benchmarking semi-reference based deconvolution methods [12]. The dataset comprised of thirty-nine samples created by systematically mixing HS-5 bone marrow stromal fibroblast cells with either wild-type HCT116 colorectal cancer cells or CRISPR-Cas9-engineered *Dicer1* knockout HCT116 colorectal cancer cells (Methods). *Dicer1* encodes an enzyme required to process most precursor microRNAs into their mature forms [45]. Knocking out *Dicer1* causes global depression of mature miRNA levels [46], which we confirmed via direct measurement of miRNA concentration per million cells (Fig. 3a). To simulate diverse tumor microenvironment scenarios, we established seven distinct miRNA mixing ratios (0:100, 30:70, 50:50, 60:40, 70:30, 85:15, and 100:0 tumor:fibroblast), with three independent biological replicates generated for each ratio. Importantly, these mixed samples were generated after measuring the total miRNA density per million cells (Fig. 3a, Methods), with extracted miRNA mixed to achieve the specified tumor-stromal total miRNA ratio, rather than cell count ratio. Furthermore, the tumor component across samples presents a large variation in total miRNA, representing either *Dicer1* wildtype or knockout cells. This design, featuring known ground truth proportions of both tumor-cell miRNA transcript and tumor-cell count and tumor cell plasticity, allowed us to thoroughly benchmark DeMixNB's accuracy across a range of scenarios typically encountered in patient-derived samples.

DeMixNB demonstrated high accuracy in predicting the true transcript proportions, achieving a root mean square error (RMSE) of 0.1 and a concordance correlation coefficient (CCC) of 0.89, with good accuracy observed across all mixing ratios (Fig. 3b, Methods). When compared to existing semi-reference-based deconvolution methods on the same dataset, DeMixNB outperformed DeMixT (RMSE = 0.22, CCC = 0.56), ISOpure (RMSE = 0.14, CCC = 0.87), and PREDE (RMSE = 0.18, CCC = 0.74), with consistently lower RMSE and higher CCC in bootstrapped resampling (adjusted pairwise Wilcoxon *p*-values < 0.001, Fig. 3b, c). Additionally, all semi-reference-based methods outperformed DeconmiR, a reference-based miRNA deconvolution method (RMSE = 0.23, CCC = 0.64) (Fig. 3b, Additional file 1: Figs. S3-4, Methods), supporting the importance of making minimal assumptions on gene expression for the tumor-cell component. These results suggest that DeMixNB can reliably estimate cell-type specific transcript proportions from bulk miRNA-seq data with greater accuracy than current methods, even in complex mixed samples with highly variable tumor specific transcripts, i.e., between *Dicer1* wildtype and knockout cells, which are deconvolved together in one batch.

### Application to bulk miRNA-seq data from breast cancer

To demonstrate the utility of tumor-specific transcript proportions of miRNA in cancer, we applied DeMixNB to a miRNA-seq dataset from 899 patients with breast cancer (BCa) in The Cancer Genome Atlas projects (TCGA) [47], where the non-tumor miRNA expression profiles can be derived from adjacent normal tissues of 98 patients (Methods, Additional file 1: Fig. S5). Prior work has established that miRNAs play key roles in breast cancer pathogenesis, where expression of oncogenic and tumor-suppressive miRNAs contribute to disease progression and therapeutic resistance [33, 48]. However, these studies have focused on individual miRNAs or on signatures built from groups of miRNAs, and as such the consequence of global tumor-cell miRNA abundance remains poorly defined. By quantifying tumor-cell miRNA proportion (π-miR), DeMixNB provides a robust framework to examine this understudied domain of tumor microRNA biology.

**Fig. 4:**
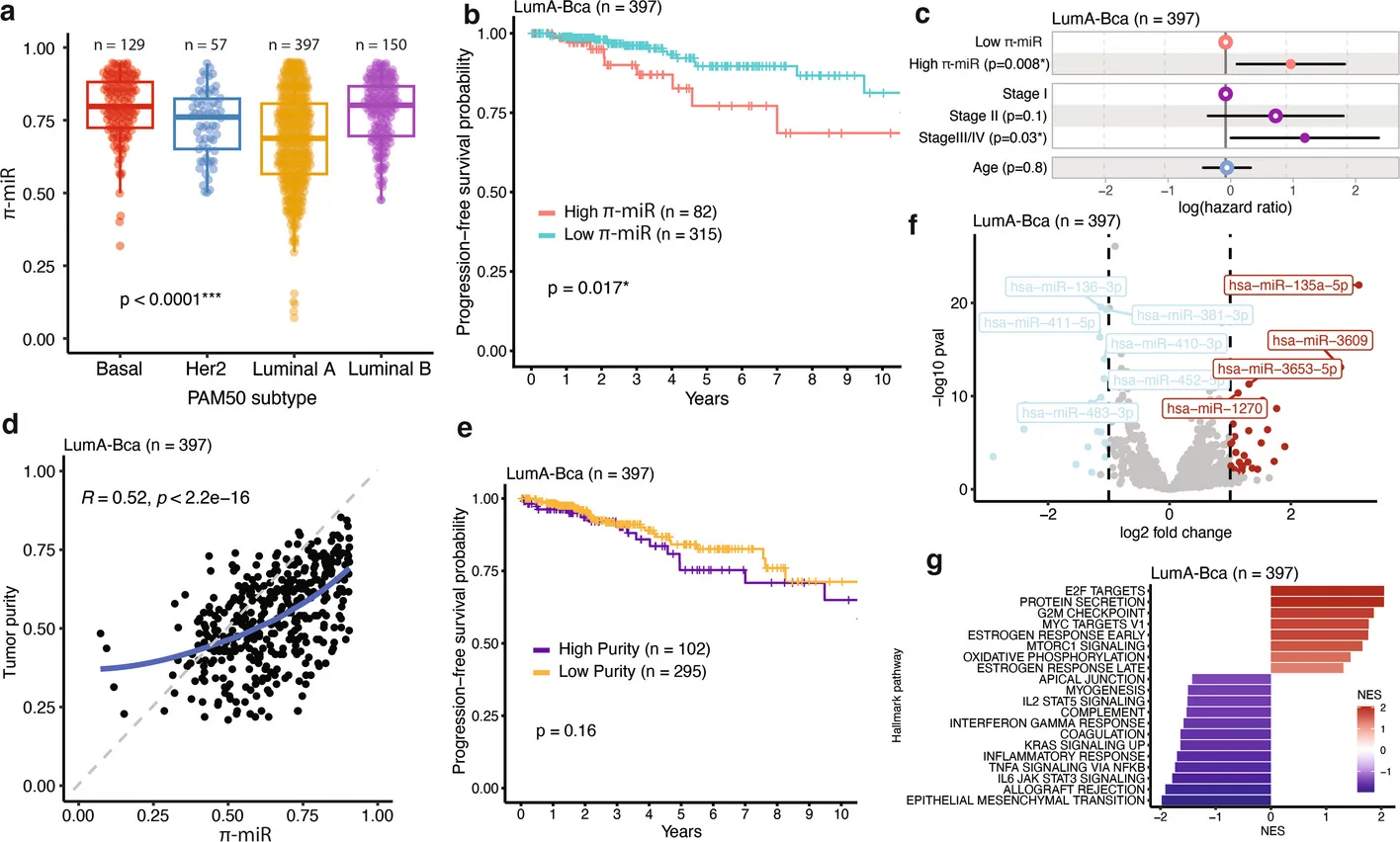
Deconvolution of bulk miRNA-seq for TCGA breast cancer. **a** Boxplots showing estimated π-miR for TCGA breast cancer patients, separated by PAM50 subtype, with the Kruskal–Wallis *p*-value shown. **b** Kaplan–Meier curves showing progression-free survival within the Luminal A breast cancer (LumA-BCa) samples, stratified by π-miR values (high vs. low, cutoff = 0.85), with the log-rank test *p*-value shown. **c** Forest plot showing hazard ratios for a Cox proportional hazard (PH) model fit on the LumA-BCa samples, with π-miR, stage, and age as covariates. Points represent the estimated hazard ratios, with 95% confidence intervals indicated by the lines. **d** Scatter plot showing relationship between π-miR and tumor purity among LumA-BCa samples. The dashed gray line indicates slope = 1, with the blue line showing the loess (locally estimated scatterplot smoothing) fit of the data. Pearson correlation coefficient is shown. **e** Kaplan–Meier curves showing progression-free survival within the LumA-BCa samples, stratified by purity values (high vs. low, cutoff = 0.66), with the log-rank test *p*-value shown. **f** Volcano plot of differentially expressed miRNAs between high and low π-miR LumA-BCa samples. The x-axis represents the log₂ fold change, and the y-axis shows the − log₁₀ adjusted *p*-value. Vertical dotted lines indicate thresholds for |log₂ fold change|> 1, and the horizontal line marks an adjusted *p*-value < 0.05 significance cutoff. **g** Bar plot summarizing significant GSEA results using genes differentially expressed between the high and low π-miR LumA-BCa samples. Hallmark pathways are ordered by normalized enrichment score, with only pathways with adjusted *p*-values less than 0.05 shown. For all plots, significance levels are denoted by: *, *p* < 0.05; **, *p* < 0.01; ***, *p* < 0.001

The deconvolved tumor-cell total miRNA proportion π-miR varied significantly across samples and PAM50 molecular subtypes, which is a classification system that stratifies breast cancers into biologically distinct groups (Luminal A, Luminal B, HER2-enriched, and Basal-like; Kruskal–Wallis *p* < 0.0001, Fig. 4a). Luminal A (LumA) breast cancers exhibited the lowest median π-miR values and showed greater heterogeneity compared to other PAM50 subtypes, indicating that the tumor total miRNA proportion may be biologically informative in this subtype (Fig. 4a). Recursive partitioning of LumA-BCa samples into high- and low π-miR groups showed that patients with high π-miR had significantly worse progression-free survival probability (PFS, log-rank *p* = 0.017, Fig. 4b, Methods). This relationship persisted in a multivariate Cox regression after adjusting for clinical stage and patient age (Fig. 4c), highlighting the independent prognostic value of π-miR. Based on our benchmark study on estimation accuracy, we chose to compare π-miR with well-benchmarked DNA copy number-based tumor cell count proportion, i.e., tumor purity estimates, instead of DeconmiR-based estimates. While π-miRs showed a moderate positive correlation with tumor purity (Methods, Pearson correlation = 0.52), they were consistently higher than the corresponding purity values, suggesting an overall increased miRNA transcriptional activity within tumor cells as compared to non-tumor cells (Fig. 4d). Finally, the recursive partitioning of tumor purity within LumA-BCa samples fails to significantly distinguish PFS (Fig. 4e, Methods), underscoring the biological and clinical information captured uniquely by π-miRs, as estimated by DeMixNB.

To understand the shifts in the miRNA landscape between high and low π-miR samples, we performed differential expression analysis of miRNAs between these groups (Methods). Among the top 10 most differentially expressed of these miRNAs (Fig. 4f), miR-411-5p, miR-410-3p, miR-381-3p, miR-136-3p, miR-483-3p have been reported to have tumor suppressive function in breast cancer [49–54], with the first four belonging to the DLK1-DIO3 miRNA cluster on chromosome 14q32, previously reported to present potential importance in many cancers [55]. The expression of miR-3609 has been shown to sensitize BCa cells to Adriamycin chemotherapy by blocking the PD-L1 immune checkpoint [56], and miR-135a-5p has context dependent roles in BCa, where it may act as a tumor suppressor by downregulating *BAG3* expression [57], with evidence also indicating that its loss promotes tamoxifen resistance in ER-positive BCa [58]. The three remaining miRNAs (miR-3653-5p, miR-1270, and miR-452-5p) have not been well characterized in BCa. The identified directions of these miRNAs in association with high or low π-miR do not always recapitulate their reported individual roles. This suggests that π-miR may capture both known and previously unexplored combined effects across all miRNAs in breast cancer.

Because of the regulatory role of miRNAs on messenger RNA (mRNA) [33], we further compared pathway-level mRNA expression profiles between high and low π-miR tumors using 405 LumA samples with matched mRNA-seq data. Gene set enrichment analysis (GSEA) revealed widespread transcriptional differences across diverse hallmark pathways, encompassing proliferative, metabolic, immune, mesenchymal and stromal processes (20 pathways had |normalized enrichment scores|> 1 and adjusted *p*-values < 0.05, Fig. 4g, Methods). Rather than implicating a single pathway or cellular function, these broad and multifaceted shifts suggest that variation in π-miR reflects a global reprogramming of gene regulation consistent with the broad influence of miRNAs on multiple targets [59]. Together, these observations indicate that π-miR captures a novel composite molecular phenotype linked to diverse biological processes within LumA breast cancer.

### Application to spatial transcriptomic data

**Fig. 5:**
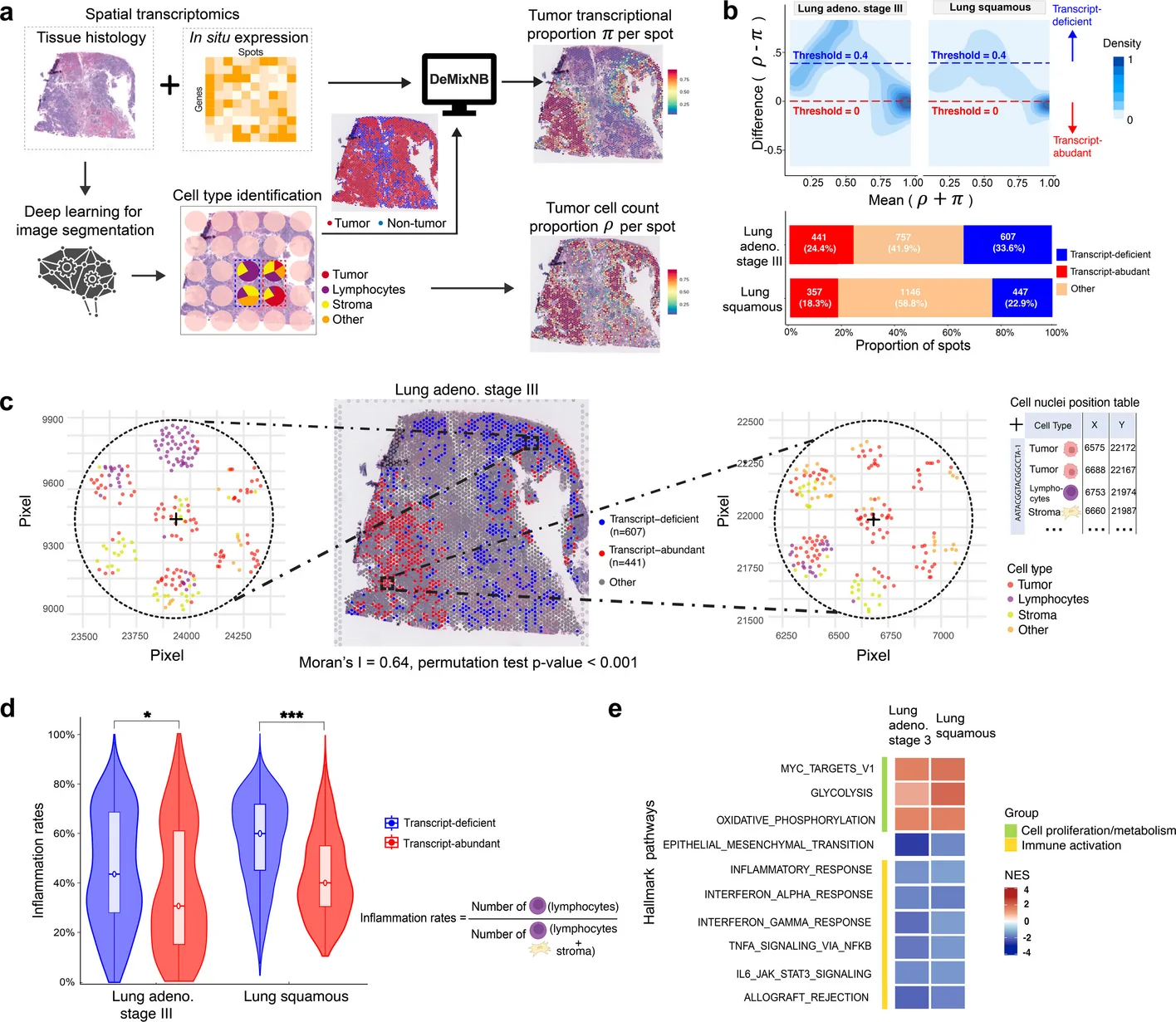
Deconvolution of spatial transcriptomics data for lung cancer. **a** Schematic illustration of the DeMixNB analytical pipeline for spatial transcriptomics data. H&E histology images undergo deep learning-based cell identification to identify tumor and multiple categories of non-tumor spots, including stromal cells and lymphocytes. Spot-level gene expression profiles from tumor-containing (mixed) spots and tumor-free (reference) spots serve as input for DeMixNB to estimate tumor transcript proportions (π). H&E-derived cell identification was also used to calculate tumor cell count proportions (ρ) for comparison. **b** MA density plots showing the distribution of $\rho -\pi$ versus their mean across spots for a lung adenocarcinoma stage III sample (left) and a lung squamous cell carcinoma sample (right). Horizontal dashed lines indicate thresholds for categorizing transcript-deficient (blue) and transcript-abundant (red) spots. Bar plots show the proportion of spots in each category for both samples. **c** Spatial visualization of transcript-deficient (blue) and transcript-abundant (red) spots overlaid on H&E histology image for the lung adenocarcinoma stage III sample. Middle panel shows Moran’s I spatial autocorrelation analysis demonstrating significant clustering (Moran’s I = 0.64, *p* < 0.001). Representative regions (left and right) highlight spots with comparable tumor cell proportions but differential transcript activities based on local immune environment. Coordinate information is used for spatial reference. **d** Comparison of local inflammation rates between transcript-deficient and transcript-abundant spots across both lung cancer samples. Inflammation rates represent the proportion of lymphocytes relative to the total lymphocyte and stromal cell count for each spot and its six adjacent neighbors. Statistical significance was assessed using the Wilcoxon rank-sum test with BH correction. **e** Gene set enrichment analysis (GSEA) results showing significantly enriched Hallmark pathways in transcript-abundant versus transcript-deficient spots. Pathways are ordered by normalized enrichment score (NES), with only pathways meeting significance thresholds (BH-adjusted *p* < 0.05) displayed. For all plots, significance levels are denoted by: *, *p* < 0.05; **, *p* < 0.01; ***, *p* < 0.001

Current spatial transcriptomics deconvolution methods primarily estimate cell-count proportions within tissue spots using single-cell RNA-seq references [60]. While proven valuable for characterizing tissue architecture and cellular neighborhoods, these approaches quantify cellular presence rather than cell-type specific transcriptional activity and thus do not capture heterogeneous transcriptional states within the TME, a distinction that is critical in cancer [7, 8, 12]. We hypothesize that the spot-level tumor cell transcript proportion may provide complementary information beyond cell counts and DeMixNB is ideal to address this question due to the sparse counts and overdispersion presented in the spot-level expression data (Additional file 1: Fig. S2). To evaluate this, we analyzed two publicly available lung cancer samples spanning distinct histology and stages—lung adenocarcinoma (LUAD) stage III [37, 38], and lung squamous cell carcinoma (LUSC) [39]. Our analytical pipeline begins with H&E slide processing using an established deep learning framework (Methods). This approach generates a cell-type count table for all spots, allowing us to catalog spots containing no tumor cells as reference and the rest as mixed tumor spots to deconvolve. After preprocessing (Methods), spot-level expression profiles for these two types of spots were used as the input data to run DeMixNB (Fig. 5a, Methods, Additional file 1: Fig. S6). Hence, DeMixNB does not require single-cell RNA sequencing (scRNA-seq) data to build reference data. DeMixNB analysis yielded tumor cell transcript proportions (π). We compared these estimates with H&E-derived tumor cell count proportions (ρ), defined as the ratio of morphologically identified malignant cells to the total number of cells detected within each spot, to assess the relationship between transcript activity and cellular proportions. This comparison was performed across 3,755 spots (with $\rho \ge 0.25$) from the 2 samples (Methods). We found H&E-derived ρ, which also does not require additional data, to be more robust across the two samples than cell count proportions estimated using public scRNA-seq as reference from RCTD [35] and CARD [36] (Methods, Additional file 1: Figs. S7-8).

To assess whether the differences between π and ρ reflect meaningful biological variation, we measured the spot-level difference (D) as $\rho - \pi$, and used the difference to identify three groups of spots (Fig. 5b, Methods): transcript-deficient spots where tumor cells maintain physical presence but exhibit substantially reduced transcriptional activity (D > 0.4), transcript abundant spots where transcript proportions meet or exceed cellular proportions (D < 0), and the rest. In the LUAD Stage III and LUSC samples, the transcript-deficient spots comprised 33.6% and 22.9% of spots, and the transcript-abundant spots comprised 24.4% and 18.3% of spots, respectively, demonstrating marked spatial variation in tumor transcriptional activity in both samples. Using Moran’s I, which measures whether different groups of spots cluster together more than expected by chance (0 = random, 1 = clustered, −1 = dispersed, Methods), we found both samples showed strong positive spatial clustering, with Moran’s I values of 0.64 (Fig. 5c) for LUAD stage III and 0.70 (Additional file 1: Fig. S9) for LUSC (permutation test *p* < 0.001 for both, Methods). This non-random spatial clustering suggests potential spatial biological factors that may regulate tumor cell transcriptional activities. Detailed examination of representative regions revealed that spots with comparable tumor cell proportions exhibited markedly different transcript activities depending on their local immune environment: a higher number of immune cells is observed in the neighborhood of transcript-deficient spots (see an example neighborhood in Fig. 5c). While recent multimodal spatial-omics study [61] has characterized the co-evolution of epithelial alveolar progenitors and proinflammatory niches in lung adenocarcinoma, the differential transcriptional response of tumor cells to this ecosystem remains to be fully quantified. We therefore further explored the relationship between immune infiltration and tumor transcript suppression by calculating a local inflammation rate across all spots. For each spot defined as transcript-deficient or abundant by $\rho - \pi$, we summed lymphocytes counts across the spot itself and its six adjacent neighbors, divided by the total lymphocytes and stromal cells in the same region to obtain a measure of local inflammation rate (Fig. 5c, Methods). Transcript-deficient spots consistently showed significantly higher inflammation rates compared to transcript-abundant spots across both samples (adjusted *p*-values < 0.05, Fig. 5d). This inverse relationship suggests that local immune pressure may actively suppress tumor cell transcript production, possibly by driving malignant cells into quiescent KRT8^+^ alveolar intermediary cells (KAC) state as proposed in recent literature [62–64]. A change of cutoff value on $\rho - \pi$ from 0.4 to 0.3 to include more transcript deficient spots replicated the above observations in both samples (Additional file 1: Fig. S10), supporting robustness of our findings. For the LUSC sample where we did not find strong evidence to rule out CARD-based ρ, we also identified transcript-deficient and transcript-abundant populations using this alternative set of tumor cell count proportion estimates and found a consistent inverse relationship between immune infiltration and transcriptional activity (Additional file 1: Fig. S11, Methods).

To validate the biological processes underlying transcript deficiency, we performed GSEA on differentially expressed genes between transcript-deficient and transcript-abundant spots in both samples (Methods). The identified pathways were highly consistent with the recently discovered inflammatory-KAC axis through multi-omic and high-resolution spatial profiling of the same sample [61]. The most prominently enriched pathways in transcript-deficient spots point to immune activation while those enriched in transcript-abundant spots correspond to cell proliferation and metabolism (Fig. 5e). Epithelial-Mesenchymal transition (EMT) was also significantly enriched in these transcript-deficient spots. The co-occurrence of high inflammation, tumor cell transcript reduction, and EMT signatures suggests that immune infiltration may induce tumor lineage plasticity, forcing malignant cells to dedifferentiate into a stress-resistant, low-transcript phenotype to survive the inflammatory microenvironment [4]. These findings demonstrate that DeMixNB-derived π captures functionally distinct tumor cell transcriptional states which are clustered across spatial regions within a tumor sample, providing an additional aspect in understanding the tumor heterogeneity in the complex TME.

## Discussion

We develop DeMixNB to address a methodological gap by enabling accurate deconvolution of sparse count sequencing data through a semi-reference-based approach with negative binomial distributions. While existing methods are primarily designed for cell-type count deconvolution, DeMixNB specifically targets tumor transcript proportion estimation and accommodates the overdispersion characteristic in miRNA-seq and spatial transcriptomics data. It is important to measure tumor transcript proportion for cancers because it provides the foundation to evaluate features of tumor cell plasticity [12]. We also generate a unique benchmark sparse-count mixed cell-line dataset with known cell count and transcript proportions to demonstrate the good performances and unique contribution of DeMixNB. Only requiring adjacent mixed non-tumor reference data rather than single-cell derived reference profiles, which are often unavailable or technically challenging to produce, DeMixNB provides a practical solution for quantifying tumor transcript proportions (π) in real-world sequencing contexts. The DeMixNB algorithm has been implemented as a function in the R package DeMixT2.0, available from the GitHub repository: https://github.com/wwylab/DeMixT, and through Bioconductor [40] at https://www.bioconductor.org/packages/release/bioc/html/DeMixT.html.

By deconvolving sparse transcripts using DeMixNB, we are able to uncover novel biological nuances when tumor cell purity (ρ) and tumor transcript proportion (π) diverge. In spatial transcriptomics data, π helps identify tumor transcript-deficient spots, where we observe non-random spatial clustering of these spots near immune-infiltrated regions, with these areas showing significantly elevated lymphocyte proportions. Although spatial tumor-immune interactions have been studied in recent spatial transcriptomic literature [37, 38, 61], DeMixNB quantifies the responsive tumor-cell specific overall transcriptional activity. Similarly, the novel prognostic utility of π-miR in TCGA breast cancer further validates this functional measure. In this scenario, elevated tumor miRNA proportions may reflect upregulation of oncogenic miRNAs or enhanced regulatory plasticity enabling cancer cell adaptability under selective pressures. Tumor total miRNA proportions represent a previously unexplored feature of cancer, and may lead to more refined understanding of the regulatory transformations of malignancy.

Future application across diverse cancer types may reveal pan-cancer trends to establish clinically robust biomarkers based on tumor transcript proportions for sparse-count RNA species. By adjusting further for tumor purity and genome dosage effects, a more accurate measure of total tumor miRNA content per tumor cell could be calculated, which may serve as a novel biomarker [12, 13]. The spatial transcriptomics capability opens critical avenues for developing region-specific signatures that can deconstruct complex bulk transcriptomic signals and quantify true tumor-intrinsic transcriptional activities. A key translational objective involves determining whether tumor-cell transcript proportions of individual spots predict clinical trajectories such as metastatic progression or immunotherapy response.

Most existing deconvolution benchmarking approaches rely on the assumption that transcript proportions directly correspond to cellular proportions, which may not fully capture cell-type-specific differences in transcriptional output and RNA content [13]. To provide a more comprehensive evaluation framework, we developed an experimental benchmarking approach that establishes ground truth based on actual transcript proportions rather than cellular ratios [12]. By measuring miRNA density per cell and mixing extracted miRNAs rather than cells themselves, we generated samples with precisely known transcript proportions. Furthermore, the inclusion of both *Dicer1* wild-type and knockout cell mixtures simulates populations with unknown tumor subtypes, reflecting the heterogeneous nature of real-world clinical samples where distinct molecular subtypes are typically uncharacterized prior to deconvolution. This approach provides a biologically grounded benchmarking standard and represents a valuable community resource for evaluating deconvolution methods across realistic scenarios.

Several limitations in DeMixNB may be addressed in future studies. The exact likelihood calculation inside the DeMixNB algorithm is more costly for computation than other methods assuming normal distributions. In simulation scenario C for 1,000 genes and 100 samples, it takes ~ 10 min for each seed run of DeMixNB (Additional file 1: Fig. S12). For a miRNA-seq data set from 100 tumor samples, it takes 2–6 h to run DeMixNB. In contrast to the longer compute time, DeMixNB demonstrated a minimal memory usage (Additional file 1: Fig. S12), indicating potential to use parallel and cloud computing for an improved balance between memory and time costs. In addition, we observe some numerical challenges during estimation due to the nature of semi-reference-based inference. We mitigate the numerical instability by running the algorithm multiple times with different starting values and generate a consensus as the final estimates. Using representative reference data is essential for running any semi-reference-based deconvolution methods including DeMixNB. A higher number of mixing components introduces a larger number of distributions to fit, hence it is more difficult to ensure goodness-of-fit of each component. In this case, the approximation of the estimated π to the tumor-cell transcript proportion (see Additional file 2) will be undermined. Hence, an attractive alternative to extending to more than 2 mixing components is to refine the gene selection approach such that the selected genes are stably expressed across most non-tumor cell types (see also simulation scenarios D and E). For more than 2 mixing components, due to the well-established challenges in estimating the overdispersion parameter [65] per gene, replacing NB with other distributions could be an option. We observed good accuracy in DeMixNB estimating the mean expressions, while the overdispersions were weakly identified (Additional file 1: Fig. S14). This was sufficient for the accurate tumor-specific transcript proportion estimation. This observation motivates switching to Poisson or Gaussian distributions that were recently successfully implemented for spatial transcriptomic deconvolution [35, 36]. The summations of these distributions are easy to manage and can be mixed with one NB distribution with further shrinkage estimation across genes [65–67] to address overdispersion. Another natural next step for spatial transcriptomic data is to develop a spatial-aware spot-level deconvolution [36] for tumor-cell transcript proportions (e.g., Tang et al. [68] developed a spatial-aware penalty). Finally, our digital pathology-derived cell count proportions were limited to four cell types, so that important immune cell populations, such as macrophages, could not be determined. This limitation restricted our current analysis from gaining deeper insights into the interactions between tumor transcripts and immune responses. Future work may incorporate more comprehensive cell type annotations using advanced digital pathology tools or complementary technologies such as multiplexed immunofluorescence. This would enable a more detailed characterization of the tumor microenvironment and a better understanding of its impact on tumor cell transcriptional activities.

## Conclusions

Modeling sparse count data such as those in spatial transcriptomics and miRNA-seq to evaluate tumor cell plasticity presents unique challenges. We developed a method, DeMixNB, that can deconvolve the tumor transcript proportions in such data without the need for single-cell-level reference data. Using a benchmarking experimental dataset, DeMixNB demonstrated better performances than existing methods. When applied to real data, DeMixNB estimates of transcript proportions capture distinct biological information not otherwise reflected in tumor purity estimates. The framework established by DeMixNB extends beyond mRNA and miRNA applications to encompass emerging bulk sequencing modalities profiling the broader RNA species, including piRNAs, snoRNAs, and lncRNAs. Therefore, DeMixNB as a tool will empower future studies interrogating the complex biology of cancer RNomes.

## Methods

### Datasets representing sparse and overdispersed expression counts

#### Generating miRNA-seq data from experimentally mixed cell-lines

We generated a benchmark dataset with known ground truth proportions. HCT116 colon cancer cells and HS-5 stromal cells were cultured at 37°C in a humidified incubator with 5% CO_2_. HS-5 cells were maintained in Dulbecco's Modified Eagle's Medium (DMEM;Corning) supplemented with 10% fetal bovine serum (FBS;Corning) and 1% Antibiotic–Antimycotic (Gibco), while HCT116 cells were maintained in McCoy’s 5A medium (Corning) with the same supplements. The *Dicer1* knockout (KO) HCT116 cells were cultured under the same conditions with the addition of G-418 selective antibiotic solution (neomycin resistance) to preserve the homogenous KO population. Total RNA was extracted from 1 × 10^6^ cells using the Zymo RNA Isolation Mini Kit (Zymo Research). Small RNA concentrations (ng/µL) were quantified using a Qubit 4 Fluorometer (Invitrogen) with the Qubit microRNA Assay Kit (Invitrogen). Based on these measurements, microRNAs from each cell type were mixed in predetermined proportions: 0:100, 30:70, 50:50, 60:40, 70:30, 85:15, and 100:0. Each set of mixtures was prepared independently for either parental HCT116 combined with HS-5 RNA or *Dicer1* KO HCT116 combined with HS-5 RNA, with three biological replicates per condition. This resulted in 5 × (3×2) + 3 × 3 = 39 samples. Sample quality was validated using an Agilent Bioanalyzer (Agilent). The miRNA sequencing libraries were prepared using the QIAseq miRNA Library Kit (Qiagen) and sequenced on an Illumina platform.

Raw sequencing data quality was assessed using FastQC, and adapter sequences were trimmed using Trim Galore. The processed reads were then aligned to miRBase (version 22.1) [69] and the human reference genome GRCh38 using the miRDeep2 [70] pipeline to quantify miRNA expression levels. Both raw data and the aggregated expression matrix for the benchmark samples have been deposited at GEO under accession number GSE311690.

#### MiRNA-seq data from patients with breast cancer

MiRNA expression data for TCGA-BRCA breast cancer samples were downloaded from the Genomic Data Commons (GDC) data portal (https://portal.gdc.cancer.gov). The miRNA count matrix was upper quartile normalized, and miRNAs detected in 10 or fewer samples were removed. Additionally, miRNAs with a mean expression greater than 10,000 in either the normal reference or mixed samples were excluded to reduce computational load and ensure adherence to the assumptions of the negative binomial model.

Breast cancer intrinsic subtype prediction was performed using the R genefu package, based on the 'PAM50' [71] subtyping signature. Transcript expression data for subtype prediction and clinical annotations (including stage and progression-free survival) were retrieved from the GDC data portal.

#### Spatial transcriptomic data from patients with lung cancer

The spatial transcriptomic data for a lung adenocarcinoma stage III sample was generated using 10x Genomics Visium technology on formalin-fixed, paraffin-embedded (FFPE) tissues at the University of Texas MD Anderson Cancer Center [37, 38]. Tissue sections (10 μm) were collected for RNA extraction using the Qiagen RNeasy FFPE Kit and quality-checked using the Agilent RNA 6000 Pico Kit (DV200 > 50%). Tissue sections were placed within capture areas (6.5 × 6.5 mm) of Visium Spatial Gene Expression Slides (PN-1000188), deparaffinized, H&E stained, and decrosslinked, followed by probe hybridization, ligation, release and extension. Libraries were constructed using the Visium Human Transcriptome Probe Kit (PN-1000363) and Visium FFPE Reagent Kit (PN-1000361) and sequenced on the Illumina NovaSeq 6000 platform (≥ 75,000 mean read pairs per spot, ≥ 2,000 median genes per spot).

The lung squamous spatial transcriptomic sample was obtained from the 10x Genomics public data repository [39] (https://www.10xgenomics.com/datasets/human-lung-cancer-ffpe-2-standard). FFPE human lung cancer tissue blocks were obtained from Avaden Biosciences. Tissue sections (5 μm) were deparaffinized, H&E-stained, decrosslinked, and processed via the Visium CytAssist instrument following demonstrated protocols (CG000518, CG000520). Libraries were constructed using the Visium CytAssist Spatial Gene Expression Reagent Kit for FFPE (CG000495) and sequenced on the Illumina NovaSeq platform (35,785 reads per spot).

### The DeMixNB model

Due to the lack of tumor reference profiles, we develop the DeMixNB model to be semi-reference-free, requiring only non-tumor expression data as well as the mixed expression data. Similar to previous deconvolution methods in the literature, we adopt the negative binomial distribution at the core of our DeMixNB model to address the overdispersion issue present in the miRNA-seq data and the spatial transcriptomics data (Additional file 1: Figs. S1-2). We focus on the deconvolution of transcript proportions in the case of two cell compartments, tumor cells and non-tumor cells, only. Denote the observed mixed expression at sample *i* for gene *g* as ${Y}_{ig}$. The deconvolution model assumes that it can be decomposed into individual contributions of each cell type, i.e. ${Y}_{ig} = {T}_{ig}+{N}_{ig}$, where ${T}_{ig}$ is the contribution of the tumor component and ${N}_{ig}$ is the contribution of the non-tumor component at sample *i* for gene *g*. Both ${T}_{ig}$ and ${N}_{ig}$ are not observed.

In contrast to the previous DeMixT model assuming log normal distributions [21], we assume that both ${T}_{ig}$ and ${N}_{ig}$ follow negative binomial distributions parameterized as ${T}_{ig}\sim NB\left({\pi}_{i}{\mu}_{tg}, {\phi}_{tg}\right)$ and ${N}_{ig}\sim NB\left(\left(1-{\pi}_{i}\right){\mu}_{ng}, {\phi}_{ng}\right)$, where ${\pi}_{i}$ represents the proportion between the tumor and non-tumor components, ${\mu}_{tg}$ and ${\mu}_{ng}$ are the means, and ${\phi}_{tg}$ and ${\phi}_{ng}$ are the dispersion parameters for ${T}_{ig}$ and ${N}_{ig}$, respectively. The expected expression of ${Y}_{ig}$ can be expressed as $E\left({Y}_{ig}\right) = E\left({T}_{ig}\right)+E\left({N}_{ig}\right) = {\pi}_{i} {\mu}_{tg} + \left(1-{\pi}_{i}\right) {\mu}_{ng}$. This is a variation that approximates the setup in previous studies [20, 21], which stated ${Y}_{ig} = {\pi}_{i} {T}_{ig} + \left(1-{\pi}_{i}\right) {N}_{ig}$. Therefore, a new assumption introduced by DeMixNB is that the mixing proportions only affect the mean expressions and do not scale with the dispersion levels across genes. This assumption deviates from real scenario but in return gains computational tractability of model parameters. In theory, if one has access to the true expressions of ${T}_{ig}$ and ${N}_{ig}$, as is the case in a simulation setting, the true transcript proportion can then be calculated as $\frac{{\sum}_{g}{T}_{ig}}{{\sum}_{g}{Y}_{ig}}$. In practice, we estimate an approximation of the true transcript proportion using estimates of the mixing proportions ${\boldsymbol{\pi}}$. This approximation has been previously proven for the model ${Y}_{ig} = {\pi}_{i} {T}_{ig} + \left(1-{\pi}_{i}\right) {N}_{ig}$ using benchmark data [12] (also see the mathematical derivation in Additional file 2). This approximation relies on data normalization between the observed mixed tumor and non-tumor samples, as well as the goodness of fit of the underlying distributions for each mixing component, and becomes more fragile as the number of components increases beyond two.

Based on the model detailed above, we implemented a DeMixNB algorithm to estimate the parameters ${\pi}_{i}$, ${\mu}_{tg}$, ${\phi}_{tg}$, ${\mu}_{ng}$, and ${\phi}_{ng}$ that maximizes the total likelihood (see a pseudo-code description of the algorithm in the Additional file 2). The input data for DeMixNB includes two data matrices: 1. ${\boldsymbol{Y}}$, the matrix containing the mixed expression data, which is the target of deconvolution; 2. ${{\boldsymbol{Y}}}^{\mathrm{r}\mathrm{e}\mathrm{f}}$, the matrix containing reference data of only non-tumor expressions. The output of DeMixNB would be the estimated transcript proportions in the mixed data ${\boldsymbol{Y}}$, as well as the other parameter estimates. We require that samples of reference data for the non-tumor component be provided, i.e. samples where ${\pi}_{i} = 0$ for all *i*, to estimate the parameters ${\mu}_{ng}$ and ${\phi}_{ng}$ first. Therefore, our method falls under the category of semi-reference-based deconvolution [13]. To estimate the remaining parameters, we take the estimated distributions of the non-tumor components as fixed, then perform an iterated estimation procedure between distribution parameters that are gene specific and mixing parameters that are sample specific until convergence. This general procedure was introduced previously as iterated conditional models (ICM [43]), and follows closely what was implemented in the DeMixT algorithm in Wang et al. [21]. ICM has been proven to guarantee convergence to local maximum, a desirable property for parameter estimation. The ICM algorithm iteratively updates the mixing proportions ${\boldsymbol{\pi}}$ and distribution parameters ${{\boldsymbol{\mu}}}_{{\boldsymbol{t,g}}}$ and ${{\boldsymbol{\phi}}}_{{\boldsymbol{t,g}}}$, maximizing the conditional likelihoods at each iteration.

The total likelihood for observing the matrix Y can be expressed as $L\left({\boldsymbol{Y}}\right)= {\sum}_{i}{\sum}_{g}L\left({Y}_{ig}\right)$, assuming the expression level for each gene *g* at each sample *i* is independent from one another. The individual likelihood of ${Y}_{ig}$ can be calculated by a convolution of the two individual probability distributions of ${T}_{ig}$ and ${N}_{ig}$. In contrast to DeMixT using numerical integration to approximate likelihood, here we can achieve an exact calculation of the total likelihood through the following formula:

$$L\left({Y}_{ig}\right) =\ \sum\limits_{z=0}^{{Y}_{ig}}{f}_{t,ig}\left(z\right){f}_{n,ig}\left({Y}_{ig}-z\right)$$

where z is the dummy variable for summation and

$${f}_{t,ig}\left(z\right) = \frac{\Gamma \left(z+{\phi}_{tg}\right)}{\Gamma \left({\phi}_{tg}\right)z!}{\left(1-{p}_{t,ig}\right)}^{z} {p}_{t,ig}^{{\phi}_{tg}}$$

$${f}_{n,ig}\left({Y}_{ig}-z\right) = \frac{\Gamma \left({Y}_{ig}-z+{\phi}_{ng}\right)}{\Gamma \left({\phi}_{ng}\right)\left({Y}_{ig}-z\right)!}{\left(1-{p}_{n,ig}\right)}^{{Y}_{ig}-z} {p}_{n,ig}^{{\phi}_{ng}}$$

$${p}_{t,ig}=\frac{{\phi}_{tg}}{{\pi}_{i} {\mu}_{tg} + {\phi}_{tg}}$$

$${p}_{n,ig}=\frac{{\phi}_{ng}}{\left(1-{\pi}_{i}\right) {\mu}_{ng} + {\phi}_{ng}}$$

*Numerical stability and software.* The DeMixNB algorithm minimizes the negative log-likelihood instead of the likelihood for improved stability. Due to the high-dimensional search space and the ICM’s converging to local minimum, we found that the initiating proportion values can result in final estimates hitting the optimizer bounds incorrectly on some occasions. They are rare but do occur for the mixture of negative binomial distributions, as compared to being negligible in the DeMixT algorithm for the mixture of log-normal distributions. Therefore, by default, our DeMixNB function will start the algorithm three times with different seeds (randomly generated starting values) and a consistency check will be performed across the runs. The user can modify the number of starting values. With each sample, if the maximum difference between the three ${\boldsymbol{\pi}}$ estimates is less than 0.1, then we deem the estimation robust. We provide the average estimates across all runs and a flag on samples with maximum difference > 0.1, for users to evaluate. Additionally, we use a quantile–quantile (QQ) analysis to identify genes for which a negative binomial fit to estimate parameters ${\mu}_{ng}$ and ${\phi}_{ng}$ does not match the observed data. In particular, we filter out genes with QQ correlations less than 0.9 before deconvolution. Finally, within each iteration, we set the bounds of the ${\boldsymbol{\pi}}$ estimates to be 0.05 and 0.95. The DeMixNB software is mainly based on the R programming language, e.g., using the built-in L-BFGS-B optimizer for parameter estimation. Computation-heavy components are implemented in C with OpenMP support to enable parallel computing across multiple threads. The DeMixNB codebase and its documentation has been integrated in the DeMixT [21] R package v2.0, available on Bioconductor [40].

### Model validation using simulated data

#### Study setup

To evaluate the performance of DeMixNB, we considered five simulated scenarios, each generating data based on summations of negative binomial distributions, with varying distributions for the mean and dispersion parameters. To generate the expression matrices, we first sample the true proportions ${\pi}_{i}$ from a uniform distribution, and then sample the mean and dispersion parameters ${\mu}_{tg}$, ${\mu}_{ng}$, ${\phi}_{tg}$, ${\phi}_{ng}$ from predefined distributions, detailed in the Simulation settings section. The mixed expression counts are subsequently generated as ${Y}_{ig}={T}_{ig}+{N}_{ig}=NB\left({\pi}_{i}{\mu}_{tg},{\phi}_{tg}\right)+NB\left(\left(1-{\pi}_{i}\right){\mu}_{ng},{\phi}_{tg}\right)$. In each scenario, 100 datasets are generated with the exact same true proportions π, and the expressions are sampled from the same negative binomial distributions with different seeds. The true transcript proportions based on the generated data, calculated as $\frac{{\sum}_{g}{T}_{ig}}{{\sum}_{g}{Y}_{ig}}$, are recorded for evaluation. With the generated data as input, we run the DeMixNB function with default settings to perform deconvolution. The estimated proportion vector is output by the algorithm for each dataset, denoted as ${\widehat{{\boldsymbol{\pi}}}}^{({\boldsymbol{d}})}$ for dataset *d*, which is compared with the true proportion vector. We also compare DeMixNB to three state-of-the-art semi-reference based deconvolution methods: DeMixT [21], ISOpure [22], and PREDE [25]. DeMixT (version 1.20.0) is capable of three-component deconvolution, but we are only using its two-component deconvolution functionality. Additionally, we did not use its built-in preprocessing and gene selection methods (by setting if.filter to FALSE) for the simulation studies. For ISOpure (version 1.1.3), we only used its first step, the ISOpure.step1.CPE function from the ISOpureR package [44] with default parameters, and extracted the output object called "alphapurities" as the estimated proportions. PREDE (version 1.2.1) requires exact values of the known expression profiles. In this case, we treated the non-tumor component as one cell type and provided the empirical means of the generated expressions as the reference expression profile. We set type = "GE", K = 2 and iters = 1000 in its function parameter to enforce two-component deconvolution.

#### Simulation settings

In all simulations, the sample size for the mixed expression matrix is set to 100 and that for the normal reference matrix is set to 50. The number of genes is fixed at 1,000. And the prior distributions for the dispersion parameters ${\phi}_{tg}$ and ${\phi}_{ng}$ are set to the same uniform distribution in scenarios A, B, and C for all genes, hence notation simplified as follows:

$${{\boldsymbol{\phi}}}_{{\boldsymbol{t}}},{{\boldsymbol{\phi}}}_{{\boldsymbol{n}}} \sim \mathrm{U}\mathrm{n}\mathrm{i}\mathrm{f}\mathrm{o}\mathrm{r}\mathrm{m}\left(0.1, 10\right)$$

We consider five different simulation scenarios with varying prior distributions for ${\mu}_{tg}$, ${\mu}_{ng}$. In scenario A, we mimic a non-sparse dataset by setting high values ${\mu}_{tg}$ and ${\mu}_{ng}$ with means around 200:

$${{\boldsymbol{\mu}}}_{{\boldsymbol{t}}},{{\boldsymbol{\mu}}}_{{\boldsymbol{n}}} \sim \mathrm{G}\mathrm{a}\mathrm{m}\mathrm{m}\mathrm{a}\left(shape=1, rate=0.005\right)$$

In scenario B, we make the data sparse by reducing the means for ${\mu}_{tg}$ and ${\mu}_{ng}$ to around 20:

$${{\boldsymbol{\mu}}}_{{\boldsymbol{t}}},{{\boldsymbol{\mu}}}_{{\boldsymbol{n}}} \sim \mathrm{G}\mathrm{a}\mathrm{m}\mathrm{m}\mathrm{a}\left(shape=1, rate=0.05\right)$$

In scenario C, we consider the case where genes might express from different distributions for the same cell type. Specifically, we consider two distributions for the tumor cells and assign 800 genes to the first distribution and 200 genes to be a mixture of both with the mixing proportion sampled uniformly between 0 and 1. Both distributions have small means to ensure the final mixed expression matrix remains sparse.

$${{\boldsymbol{\mu}}}_{{\boldsymbol{t}}1},{{\boldsymbol{\mu}}}_{{\boldsymbol{n}}} \sim \mathrm{G}\mathrm{a}\mathrm{m}\mathrm{m}\mathrm{a}\left(shape=1, rate=0.05\right)$$

$${{\boldsymbol{\mu}}}_{{\boldsymbol{t}}2} \sim \mathrm{G}\mathrm{a}\mathrm{m}\mathrm{m}\mathrm{a}\left(shape=1, rate=0.1\right)$$

In the next two scenarios, we are focused on evaluating the impact of assuming one single negative binomial (NB) distribution on the heterogeneous non-tumor component. As we observed a wide range of mixed immune and stromal cells from the H&E estimated proportions in the spatial transcriptomic (ST) data, we simulated the parameters following closely the observed ST data, hence using different distributions from previous scenarios.

In scenario D, we consider the case where genes might express differently for a mixture of cell types in the non-tumor component. Similar to scenario C, we consider two negative binomial distributions for the non-tumor cells. We let 200 genes express based on a single negative binomial distribution and the remaining 800 to be a mixture of two negative binomial distributions, with the mixing proportion sampled uniformly between 0 and 1. In this scenario, the mixture of two distributions can still be approximated by a single negative binomial distribution (Additional file 1: Fig. S13).

$${{\boldsymbol{\mu}}}_{{\boldsymbol{t}}},{{\boldsymbol{\mu}}}_{{\boldsymbol{n}}1},{{\boldsymbol{\mu}}}_{{\boldsymbol{n}}2} \sim {e}^{Exponential\left(rate=2\right)}$$

$${{\boldsymbol{\phi}}}_{{\boldsymbol{t}}},{{\boldsymbol{\phi}}}_{{\boldsymbol{n}}1},{{\boldsymbol{\phi}}}_{{\boldsymbol{n}}2}\sim \mathrm{G}\mathrm{a}\mathrm{m}\mathrm{m}\mathrm{a}\left(shape=4.44, rate=4.8\right)$$

In scenario E, we make the distributions of the non-tumor component bimodal to explicitly violate the negative binomial assumption in a subset of genes. Specifically, we let 800 of the genes express based on a single negative binomial distribution with the same hyperparameters as scenario D. The remaining 200 genes are expressed based on a mixture of the negative binomial distribution and a normal distribution with a mixing proportion of 0.5 for each, thus creating a non-negative-binomial, bimodal distribution.

$${{\boldsymbol{\mu}}}_{{\boldsymbol{t}}},{{\boldsymbol{\mu}}}_{{\boldsymbol{n}}1}\sim {e}^{Exponential\left(rate=2\right)}$$

$${{\boldsymbol{\phi}}}_{{\boldsymbol{t}}},{{\boldsymbol{\phi}}}_{{\boldsymbol{n}}1}\sim \mathrm{G}\mathrm{a}\mathrm{m}\mathrm{m}\mathrm{a}\left(shape=4.44, rate=4.8\right)$$

$${{\boldsymbol{\mu}}}_{{\boldsymbol{n}}2}\sim {e}^{Exponential\left(rate=20\right)}$$

$${{\boldsymbol{\sigma}}}_{{\boldsymbol{n}}2}\sim \mathrm{G}\mathrm{a}\mathrm{m}\mathrm{m}\mathrm{a}\left(shape=4.44, rate=4.8\right)$$

In scenarios D and E, we tested two ways of running PREDE: 1). providing the empirical means of the mixed reference data as partial reference and performing two-component deconvolution; 2). providing the means of each component of the mixed reference data and performing three-component deconvolution. The first approach generated better results, which were included in the comparison. This is likely because providing the mean parameters for more mixing components amplifies the violation of the Gaussian noise assumption in PREDE.

To evaluate the performance of the model, we calculate the average absolute bias across the 100 repeats, the average root mean squared error (RMSE), and the average concordance correlation coefficient (CCC) between the estimated and the true proportions. For each dataset, we also calculate a consistency metric (sumEst) where we use the parameters for the tumor component to generate reference data to estimate the proportion of the non-tumor component and verify that the sums of the estimates for the non-tumor component and those for the tumor component are close to 1.

### Model validation using experiment data as benchmark

#### Experimental setup

In our benchmark dataset, we have reference profiles for the pure fibroblasts, *Dicer1* wildtype cancer cells, and *Dicer1* knockout cells. We benchmarked deconvolution performance for all methods using all samples mixed from both the wildtype and knockout cells, i.e., the tumor-specific total miRNA transcripts vary largely across samples, as this represents a realistic biological scenario: cancers in a cohort may come from distinct subtypes with different expression profiles. We evaluated DeMixNB against three established semi-reference-based deconvolution methods, DeMixT, ISOpure, and PREDE, as well as a reference-based deconvolution method for miRNA-seq, DeconmiR [34]. Prior to deconvolution analysis, the miRNA count matrix was normalized using upper quantile normalization. The normalized expression matrix was then used as input for all methods.

#### Deconvolution of benchmarking data

For DeMixNB, pure HS-5 fibroblast samples (MIX-1–1, MIX-2–1, MIX-3–1) were used as the reference normal component. The remaining mixed samples were treated as the input bulk tissue samples requiring deconvolution. DeMixT version 1.20.0 was used with parameters set to gene.selection.method = "GS", ngene.Profile.selected = 200, ngene.selected.for.pi = 200, niter = 65, nbin = 50, and filter.sd = 2. For PREDE (version 1.2.1), we implemented the PREDE function with parameters type = "GE", K = 2 (representing two cell types), and iters = 1000, using the average expression profile of the pure HS-5 samples as the reference normal component. For ISOpure (version 1.1.3), the ISOpure.step1.CPE function from the ISOpureR package [44] was run using default parameters. For DeconmiR [34] (version 0.0.0.9000) since the model requires a full set of reference profiles for both T and N, we set up the T component in three ways: 1) the average expression of *Dicer1* wildtype and knockout cell lines for all mixed samples; 2) the average expression of one type of tumor cells: wildtype for all, knockout for all; 3) the average expression of matched tumor cell types: wildtype for wildtype, knockout for knockout. These settings deviate further and further away from real data scenarios for deconvolution. Deconvolution was performed using (method = "RPC").

Model performance was evaluated by calculating the root mean square error (RMSE) and concordance correlation coefficient (CCC) between the ground truth mixing proportions and the estimated tumor proportions from each method. SumEst was not used in this evaluation, as a single reference profile for both cancer cell populations is feasible. We used bootstrapping to measure uncertainty. From the mixed samples, we randomly sampled sets of 36 samples from the mixed-cell benchmarking dataset, 100 times with replacement. If a sampling didn’t include 3 different mixing proportions, this sample would be redrawn. The Wilcoxon rank-sum test was used to assess the differences between paired CCC and RMSE distributions from the above methods with Benjamini-Hochberg (BH) correction [72].

### Analysis of TCGA-BCa miRNA-seq data

#### Deconvolution

Deconvolution of tumor miRNA proportions was carried out for 899 TCGA breast cancer samples, using matched normal tissues from 98 samples as normal reference. Prior to deconvolution, input count matrices were upper-quartile normalized, and miRNAs with mean expression across samples > 10,000 were filtered out. Additionally, miRNAs that were poorly fit using a negative binomial model in the normal samples (QQ correlation < 0.9) were removed (Additional file 1: Fig. S5), resulting in a set of 1,079 miRNAs used for deconvolution. Following deconvolution, samples with unstable estimates across three initial values were removed (*n* = 885, with 14 samples removed), and samples were further filtered to those with clinical annotations including stage and PAM50 subtypes (*n* = 733).

#### Comparison with tumor purity estimates

ASCAT [73]-based tumor purity values were obtained from its GitHub repository (https://github.com/VanLoo-lab/ascat/tree/master/ReleasedData). 

#### Survival analysis

Within the Luminal A subgroup, tumor purity–associated differences in progression-free survival were evaluated using recursive partitioning, Kaplan–Meier analysis with log-rank tests, and Cox regression. Recursive partitioning was performed with the rpart package (v4.1.23) to identify an optimal cutoff for π-miR and ASCAT-based tumor purity using parameters that restricted the tree to a single split (maximum depth = 1), required a minimum of five observations to attempt a split, a minimum terminal node size of 10% of the samples, and a complexity parameter set to a low value (cp = −0.01) to allow the model to identify a single, unbiased cutoff point without pruning additional splits. Progression-free survival differences between the resulting high π-miR and low π-miR groups, along with high and low ASCAT-derived purity, were assessed using Kaplan–Meier analysis with log-rank tests. A multivariate Cox proportional hazards model incorporating age and the clinical pathological stage was also used to evaluate the independent prognostic value of π-miR.

#### Differential expression and gene set enrichment analysis

Differential expression analysis of miRNA and mRNA transcripts was performed using DESeq2 (v1.44.0). Gene set enrichment analysis (GSEA) of differentially expressed genes was conducted using fgsea (1.30.0) with genes ranked by normalized enrichment scores (NES) from DESeq2. Hallmark pathway gene sets were obtained from the Molecular Signatures Database (https://www.gsea-msigdb.org/gsea/msigdb/collections.jsp).

### Deconvolution of spatial transcriptomic data

#### Deep learning-based cell classification

Cell detection and classification were achieved using a deep learning pipeline previously validated in lung cancer [74, 75]. Initially, the whole-slide H&E images at full resolution were segmented using Micro-Net [76] to remove background noise and artifacts. Subsequently the nuclei within the segmented tissue regions were identified using a Spatially Constrained Convolutional Neural Network (SCCNN) model [77] for cell detection. Cell classification was performed using a neighboring ensemble predictor and voting strategy. A tumor microenvironment segmentation model [75] was then applied to generate semantic segmentation maps to delineate tumor regions. This provides additional contextual information to reduce false positive tumor cell detections. With this pipeline, cells in the tissue are categorized into four groups: tumor cells (malignant epithelial cells), lymphocytes (including plasma cells), non-inflammatory stromal cells (fibroblasts and endothelial cells), and an ‘other’ category that includes non-identifiable, and less abundant cells such as macrophages. The pipeline outputs a table with cell locations and types, enabling the linkage of individual cells to spots. Using these data, the tumor cell count proportion (ρ) for a given spatial spot is defined as the absolute count of malignant epithelial cells divided by the total count of all cells detected within the boundaries of that spot.

#### Data pre-processing

Raw spatial transcriptomic data were preprocessed to ensure data quality and suitability for downstream analyses. Initial gene filtering removed mitochondrial genes (MT-), microRNA genes (MIR-), long intergenic non-coding RNAs (LINC), immunoglobulin genes (IG*), and T-cell receptor genes (TR*). Genes detected in fewer than 10 spatial spots were excluded. Subsequently, spatial spots were filtered based on minimum library size (≥ 600 total unique molecular identifiers, UMIs) and gene detection thresholds (≥ 300 detected genes). H&E-based cell-type proportions (tumor, lymphocytes and stroma) and cell counts per spot are summarized and used to identify tumor-mixed and non-tumor spots. Spots containing fewer than five total cells were excluded. Spots without tumor cells identified through this pipeline served as reference spots for subsequent tumor-specific deconvolution. After filtering, we have 2,252 mixed spots and 834 reference spots for the LUAD stage III sample and 2,457 mixed spots and 1,214 reference spots for the LUSC sample for deconvolution.

#### Gene selection for deconvolution

We first normalized and scaled the spatial transcriptomics data using the SCTransform function from the Seurat R package (v4.0). Principal component analysis (PCA) was performed on the resulting SCT assay data with the RunPCA() function. Graph-based clustering FindNeighbors() and FindClusters(), resolution = 0.8) and uniform manifold approximation and projection (UMAP; RunUMAP()) embedding were subsequently performed using the first 50 principal components. Spatially variable genes were then identified using the Moran's I statistic (FindSpatiallyVariableFeatures(), selection method = "moransi"), computed on all genes identified by SCTransform. Genes were ranked according to observed Moran's I values, and the top-ranked genes (~ 1,000 genes) were selected for deconvolution. Among these, 15 genes whose count distributions poorly fit the negative binomial model (QQ correlation < 0.9 for the observed counts against a fitted NB distribution) were excluded (Additional file 1: Fig. S6), leaving 978 genes for LUAD and 985 genes for LUSC.

#### Reference-based deconvolution for tumor cell count proportions

To obtain reliable estimates of tumor cell count proportions, we compared the H&E-based estimates with widely used single-cell reference-based deconvolution (e.g., RCTD [35], CARD [36]) derived proportions. The raw count matrices were preprocessed using the same preprocessing pipeline as for DeMixNB. Single-cell reference datasets were obtained from previous studies [78, 79]. Both reference datasets were preprocessed to retain well-annotated cells across samples and the reference profiles were built using 10,210 cells across 14 cell types for LUAD and 3,514 cells across 9 cell types for LUSC. For CARD, we followed the standard tutorial on GitHub to create CARD objects with default parameters. For RCTD, we created spatialRNA objects with max_cores = 4, doublet_mode = ’full’. For both methods, the spot-level malignant cell proportion served as the tumor cell count proportion ρ in the comparison with H&E-based estimates.

#### Spot grouping

We obtained the transcript proportion estimates for π for 2,252 and 2,457 spots for the LUAD stage III and LUSC samples, respectively. We selected thresholds for classifying spots as tumor transcript-deficient or tumor transcript-abundant by comparing digital pathology-derived tumor cell count proportion (ρ) with DeMixNB-derived tumor cell transcript proportion (π). To ensure robust analysis, we restricted our selection to spots with at least moderate tumor content (H&E estimated $\rho \ge 0.25$), as difference D in regions with very low tumor purity are difficult to interpret biologically and may reflect measurement noise rather than real transcriptional differences. After this filtering, we obtained 1,805 spots from LUAD stage III sample and 1,950 spots from LUSC sample. We defined transcript-deficient spots as those with D>0.4 (or 0.3 for sensitivity analysis), where ρ substantially exceeded π, and transcript-abundant spots as those with D<0, where π exceeded ρ. These thresholds were chosen based on the observed density distribution patterns to capture biologically distinct populations while maintaining sufficient sample sizes for downstream statistical analysis.

#### Spatial clustering analysis and permutation test

To assess spatial clustering of transcript-deficient and transcript-abundant spots, we computed Moran’s I on the binary group label using row-standardized spatial weights (nb2listw, style = “W”, zero.policy = TRUE) derived from an adaptive neighbor graph constructed by distance-based criteria with k-nearest-neighbor fallback. Moran’s I measure spatial autocorrelation ranging from −1 (perfect dispersion) to 1 (perfect clustering), with 0 indicating random spatial distribution. Statistical significance was assessed by a Monte Carlo permutation test (moran.mc, nsim = 10000). Under the null hypothesis of spatial randomness, group labels were randomly reassigned across spots while maintaining the spatial weight structure. This process was repeated 10,000 times to generate an empirical null distribution of Moran’s I values. The one-sided *p*-value was calculated as the proportion of permuted statistics greater than or equal to the observed value, testing for positive spatial autocorrelation. An observed Moran’s I consistently exceeding all permuted values indicates statistically significant spatial clustering of spot groups.

#### Spot-specific inflammation rate

To quantify local tumor microenvironment composition, we calculated an inflammation rate representing the abundance of lymphocytes versus stromal cells within each spatial transcriptomic spot and its immediate neighbors. We determined spatial adjacency between spots based on Euclidean distance of their coordinates, identifying neighbors within a predefined threshold (140 μm). For each target spot, the total stromal and lymphocyte counts were summed across the target spot and its neighboring spots (6 spots), forming a localized pseudo-bulk composition. The inflammation rate was computed as

$$Inflammation\ rates = \frac{number\ of\ lymphocytes}{number\ of\ stromal\ cells + number\ of\ lymphocytes}$$

Spots with zero total stromal and lymphocytes cells were excluded from scoring to avoid division by zero.

The calculated inflammation rates were then compared statistically between two defined spot groups (“transcript-deficient” and “transcript-abundant”) using the Wilcoxon rank-sum test implemented with the wilcox.test() function with BH correction in R.

## Supplementary Information

Additional file 1: Supplementary tables and figures supporting the main text, including: simulation results; characterization of miRNA vs. mRNA expression ranges and overdispersion; DeconmiR performance under Dicer1 wildtype/knockout references; QQ-correlation filtering diagnostics for miRNA-seq and spatial gene expression; comparisons of tumor-cell count and transcript proportions across deconvolution and digital pathology methods; spatial clustering and inflammation analyses of transcript-deficient vs. transcript-abundant spots; and DeMixNB runtime, memory, and expression parameter estimation.

Additional file 2: Mathematical derivation for approximating tumor-specific transcript proportions, and pseudocode description of DeMixNB algorithm.

## Data Availability

MiRNA and mRNA expression data, along with clinical annotations for TCGA-BRCA breast cancer samples were downloaded from the Genomic Data Commons (GDC) data portal (https://portal.gdc.cancer.gov). Benchmarking dataset files and aggregated expression matrix have been deposited at GEO under accession number GSE311690 [80]. The DeMixNB algorithm has been implemented as a function in the R package DeMixT2.0, available from the DeMixT [81] GitHub repository under a GPL-3.0 license: https://github.com/wwylab/DeMixT, and through the Bioconductor DeMixT package [82] at https://www.bioconductor.org/packages/release/bioc/html/DeMixT.html. A snapshot of the package version used to generate these results has been deposited at Zenodo under doi: https://doi.org/10.5281/zenodo.19897616 [83]. Both spatial transcriptomic datasets are publicly available and previously published. The lung adenocarcinoma stage III spatial transcriptomic data was accessed from the Gene Expression Omnibus (GEO) under the accession number GSE307534 [61, 84]. The 10X Visium lung squamous cancer data [39] is available at the 10X website. The sample overview is available at https://www.10xgenomics.com/resources/datasets/human-lung-cancer-ffpe-2-standard, with links for file downloads available there, including the raw bam file link: https://cf.10xgenomics.com/samples/spatial-exp/2.0.0/CytAssist_FFPE_Human_Lung_Squamous_Cell_Carcinoma/CytAssist_FFPE_Human_Lung_Squamous_Cell_Carcinoma_possorted_genome_bam.bam.
