# Supplementary material for: DeMixNB: deconvolution of sparse-count RNA sequencing data for tumor cells using embedded negative binomial distributions
Source: Genome Biol. 2026 Aug 10;27:347. doi: 10.1186/s13059-026-04234-4 (PMC13741493; doi:10.1186/s13059-026-04234-4)
Supplement: Supplementary file 1 [file 13059_2026_4234_MOESM1_ESM.docx]

| Scenario | Model | Bias | RMSE | CCC | sumEst |
| --- | --- | --- | --- | --- | --- |
| A: Non-sparse data  G=1000  $S_{t}$=100  $S_{n}$=50 | DeMixNB | **0.021** | **0.029** | **0.979** | **1.029** |
|  | DeMixT | 0.038*** | 0.042*** | 0.974 | 1.098*** |
|  | ISOpure | 0.136*** | 0.142*** | 0.790*** | 1.281*** |
|  | PREDE | 0.089*** | 0.098*** | 0.884*** | 1.207*** |
| B: Sparse data with one tumor distribution  G=1000  $S_{t}$=100  $S_{n}$=50 | DeMixNB | **0.020** | **0.024** | **0.982** | **1.032** |
|  | DeMixT | 0.055*** | 0.060*** | 0.947*** | 1.116*** |
|  | ISOpure | 0.152*** | 0.158*** | 0.750*** | 1.310*** |
|  | PREDE | 0.088*** | 0.097*** | 0.885*** | 1.207*** |
| C: Sparse data with two tumor distributions  G=800,200  $S_{t}$=100  $S_{n}$=50 | DeMixNB | **0.018** | **0.022** | **0.989** | **1.023** |
|  | DeMixT | 0.061*** | 0.067*** | 0.954*** | 1.114*** |
|  | ISOpure | 0.139*** | 0.147*** | 0.830*** | 1.276*** |
|  | PREDE | 0.133*** | 0.163*** | 0.769*** | 1.260*** |
| D: Sparse data with two non-tumor distributions  G=800,200  $S_{t}$=100  $S_{n}$=50 | DeMixNB | **0.117** | **0.151** | **0.686** | 1.168 |
|  | DeMixT | NA | NA | NA | NA |
|  | ISOpure | 0.469*** | 0.511*** | 0.000*** | **0.923***** |
|  | PREDE | 0.173*** | 0.213*** | 0.595*** | 1.185 |
| E: Bimodal non-tumor distributions  G=800,200  $S_{t}$=100  $S_{n}$=50 | DeMixNB | **0.191** (0.291) | **0.225** (0.335) | **0.468** (0.169) | 1.222 (1.307) |
|  | DeMixT | NA | NA | NA | NA |
|  | ISOpure | 0.329*** (0.341)*** | 0.386*** (0.397)*** | 0.000*** (0.000)*** | 1.542*** (1.556)*** |
|  | PREDE | 0.332*** (0.334)*** | 0.375*** (0.376)*** | 0.274*** (0.271)*** | **1.200** (1.208) |

**Supplementary Tables**

**Table S1. Simulation study.** *G* represents the number of genes per sample. $S_{t}$ and $S_{n}$ denote the number of mixed samples and normal reference samples, respectively. Paired t-tests have been conducted between DeMixNB and each of the other three methods and the Benjamini-Hochberg-adjusted-p-values for each metric are reported (significance level ***: p < 0.001, **: p < 0.01, *: p < 0.05). The best performing model for each metric is bolded. For scenarios D and E, the values in the parentheses are results from running the methods without gene selection. Note that DeMixT did not produce meaningful results for scenarios D and E, and are therefore omitted.

**Supplementary Figures**

**Fig S1. Comparison of miRNA and mRNA expression dynamic ranges in TCGA-BRCA. Density distributions of mean expression for miRNA (blue, n = 1,227 features) and mRNA (orange, n = 18,146 features) across matched TCGA-BRCA tumor samples. Mean expression was computed on size-factor normalized counts (DESeq2**^1^ **median-of-ratios normalization) and is shown on a log scale. Dotted vertical lines indicate the median per modality (miRNA: 1.5; mRNA: 1,017 normalized counts). mRNA was pre-filtered using the standard bulk RNA-seq expression filter (edgeR**^2^ **filterByExpr, min.count = 10, min.total.count = 15) to retain expressed genes; miRNA was prefiltered to remove features with > 95% zeros across samples. The two modalities span overlapping but markedly different dynamic ranges: typical mRNA expression is approximately two orders of magnitude higher than typical miRNA expression, and the mRNA distribution is more concentrated around its mode. This difference in expression scale and distributional shape motivates modality-specific count modeling choices for downstream deconvolution and differential expression analyses.**

**Fig S2. Evaluation of overdispersion for miRNA-seq and spatial transcriptomics data**. (a) Mean–variance relationship for miRNA expression in the benchmarking dataset. Each point represents one miRNA's empirical mean and variance across samples (size-factor normalized counts, log–log scale). The red solid line shows the Poisson expectation (σ² = μ); the blue dashed line shows the fitted Negative Binomial (NB) variance trend, σ² = μ + α(μ)·μ², where α(μ) is the parametric mean–dispersion trend estimated by DESeq2. miRNA expression shows substantial overdispersion relative to Poisson across the full expression range, with variance scaling approximately quadratically with the mean at higher expression levels. (b) Distribution of per-miRNA NB dispersion estimates (α) for the benchmarking dataset, shown on a log scale. Values are DESeq2 maximum-a-posteriori (MAP) shrunken estimates, which are more reliable than per-gene maximum-likelihood estimates for modest sample sizes. The blue dotted line marks the median fitted dispersion. A Poisson model would correspond to α ≈ 0 (off-axis to the left on the log scale); the observed distribution sits well above this regime, indicating that Poisson cannot capture the biological and technical variability in miRNA-seq data. (c, d) Same analyses as (a, b) for 1,231 miRNAs across 899 breast cancer (BRCA) samples from TCGA. The pattern of overdispersion is consistent with the benchmarking dataset, with comparable median dispersion. (e, f) Same analyses as (a, b) for spatial transcriptomics expression of 16,356 genes across 3,671 spots (2,457 tumor, 1,214 normal) from a LUSC sample. Despite the much wider dynamic range of mRNA expression relative to miRNA, the same NB variance structure holds, demonstrating that the overdispersion phenomenon is not specific to miRNA data and that a NB-based count model is appropriate for both modalities.

**Fig S3. Comparison of DeconmiR performance when using *Dicer1* wildtype (left) or *Dicer1* knockout (right) as reference for all mixed samples.**

**Fig S4. Comparison of DeconmiR performance when using *Dicer1* wildtype to deconvolve wildtype mixtures (left) or *Dicer1* knockout to deconvolve knockout mixtures (right). This deconvolution procedure requires knowing *a priori* which mixed samples are based on *Dicer1* wildtype and which ones are based on *Dicer1* knockout.**

**Fig S5. Histogram of QQ correlation coefficients between observed and theoretical quantiles for miRNA-seq for adjacent normal samples in TCGA breast cohort. Blue vertical dashed line indicates 0.9 cutoff used for inclusion in deconvolution. 317 miRNAs did not produce valid correlation coefficients due to extreme sparsity. For sake of visualization, these miRNAs are given correlation = 0. A total of 1,079 miRNAs are retained after filtering out those with correlation < 0.9.**

**Fig S6. Histogram of QQ correlation coefficients between observed and theoretical negative binomial quantiles for gene expression in reference spots of the LUAD (left) and LUSC (right) samples. Blue vertical dashed line indicates the QQ correlation = 0.9 cutoff. Genes with a QQ correlation < 0.9 were excluded from deconvolution. A total of 13,695 and 16,341 genes were retained for the LUAD and LUSC samples, respectively, after filtering.**

**Fig S7. Pairwise comparison of tumor cell count proportions and tumor cell transcript proportions from deconvolution and digital pathology methods for a LUAD sample (left) and a LUSC sample (right). Pairwise scatter plot matrix comparing estimates from** $\rho_{CARD}$**,** $\rho_{RCTD}$**,** $\rho_{Digital Histology}$ **and** $\pi$**. The lower-diagonal panels show pairwise scatter plots with lowess() smoothing curves (red). The upper-diagonal panels display the Pearson correlation coefficients (r) and the Concordance Correlation Coefficient (CCC). The diagonal panels show the probability density distribution of estimates for each respective method. The moderate correlation demonstrated by** $\rho_{Digital Histology}$ **and** $\pi$ **between 0.52 and 0.62 matches what is observed in our miRNA-seq deconvolution study. On the other hand, even though** $\rho_{CARD}$ **and** $\rho_{RCTD}$ **are strongly correlated with each other, their correlations with** $\pi_{T}$ **are at a moderate level for LUSC with balanced deviations on both sides of the smoothed curve. This is not the case for LUAD. Importantly in LUAD,** $\rho_{CARD}$ **and** $\rho_{RCTD}$**identified a lot more spots as almost 100% tumor cells whereas** $\rho_{Digital Histology}$ **reported a wide range of proportions. Hence we investigated these spots in Fig S8.**

**Fig S8. Validation of digital pathology-based tumor cell count proportions versus CARD in the lung adenocarcinoma stage III sample. (a) Representative region showing discordance between CARD-derived and digital pathology-based tumor cell count proportions.** Blue circles in the tumor core represent spots where the two methods align (difference < 0.1), red circles at the boundary represent spots where the two methods differ substantially (difference > 0.3). (b) Cell-type annotation of the same region showing tumor cells (green), lymphocytes (blue), stromal cells (yellow), and other cell types (gray). Digital pathology estimates ($\rho_{p}$) showed better consistency with the observed histological patterns, particularly in boundary regions with mixed cell populations.

**Fig S9. Spatial clustering of transcript-deficient and transcript-abundant spots for the lung squamous sample.** Spatial visualization of transcript-deficient (blue) and transcript-abundant (red) spots overlaid on H&E histology image. Moran’s I spatial autocorrelation analysis demonstrates significant clustering (I=0.70, permutation test p<0.001).

**Fig S10. Comparison of local inflammation rates between transcript-deficient and transcript-abundant spots selected using an alternative difference threshold of 0.3.** Inflammation rates represent the proportion of lymphocytes relative to the total lymphocyte and stromal cell count for each spot and its six adjacent neighbors. Lowering the threshold from 0.4 to 0.3 increased the number of transcript-deficient spots, replicating our findings in both samples. Statistical significance was assessed using Wilcoxon rank-sum test with Benjamini-Hochberg (BH) correction. Significance level is denoted by: *, p < 0.05; **, p < 0.01; ***, p < 0.001; ****, p < 0.0001.

**Fig S11. Validation of DeMixNB findings using CARD-derived tumor cell count proportions for the lung squamous sample. (a)** MA density plot showing the distribution of differences ($D= \rho-\pi$) between tumor transcript proportions and CARD-derived tumor cell count proportions versus the means. Horizontal dashed lines indicate thresholds for categorizing transcript-deficient ($D\geq0.4$, blue) and transcript-abundant ($D\leq0$; red) spots. **(b)** Bar plot shows the proportion of spots in each category. **(c)** Comparison of local inflammation rates between transcript-deficient and transcript-abundant spots. Inflammation rates represent the proportion of lymphocytes relative to the total lymphocyte and stromal cell count for each spot and its six adjacent neighbors. Statistical significance was assessed using Wilcoxon rank-sum test with Benjamini-Hochberg (BH) correction. Significance level is denoted by: *, p < 0.05; **, p < 0.01; ***, p < 0.001; ****, p < 0.0001.

**Fig S12. Runtime and memory usage benchmarking in simulation scenario C.** Both runtime and peak memory usage are measured using the peakRAM R package (version 1.0.2) in 100 repeated runs. Runtime measures the time taken to complete the function call for each method and is shown in seconds and minutes for visualization. Peak memory refers to the maximum memory usage within the function call and is displayed in log_10_ scale MiB (Mebibyte, 2^20^ bytes).

**Fig S13. Distribution of correlation coefficients between observed and theoretical quantiles for one run in simulation scenario D.** Blue vertical dashed line indicates 0.9. Out of the 1,000 genes in the simulation and across 100 runs, almost all genes have a QQ correlation of over 0.9, indicating that a single negative binomial distribution can fit the mixed reference data well.

**Fig S14. Mean expression estimation by DeMixNB against the true values in both simulation and benchmark data.** In simulation scenario C, the true $\mu_{t}$ for genes with two tumor components is calculated by the weighted sum of the two mean parameters where the weight is the mean of the proportions across all samples. In the benchmark data from mixing miRNAs, gene selection was applied to remove genes that do not fit the negative binomial distribution based on the QQ-correlation. There were a few unusually high estimates (0.7% of all genes) in simulation scenario C and are represented as triangles at the top. This is purely a choice to maintain the x-axis limit and the aspect ratio. There is no bounding during estimation. This issue did not occur with the benchmark data. While $\mu_{t}$ can be relatively well-estimated with Spearman correlations of 0.965 and 0.956 respectively, DeMixNB’s performance is weaker on the overdispersion parameter $\phi_{t}$, with Spearman correlations of 0.568 and 0.503 respectively. With reasonable estimates of the expression distribution parameters, DeMixNB achieved high accuracy in proportion deconvolution in simulation scenario C and the benchmark study, with CCC values of 0.99 and 0.89 respectively.

**References**

1.	Love, M. I., Huber, W. & Anders, S. Moderated estimation of fold change and dispersion for RNA-seq data with DESeq2. *Genome Biol.* **15**, 550 (2014).

2.	Chen, Y., Chen, L., Lun, A. T. L., Baldoni, P. L. & Smyth, G. K. edgeR v4: powerful differential analysis of sequencing data with expanded functionality and improved support for small counts and larger datasets. *Nucleic Acids Res.* **53**, gkaf018 (2025).
