# Supplementary material for: DeMixNB: deconvolution of sparse-count RNA sequencing data for tumor cells using embedded negative binomial distributions
Source: Genome Biol. 2026 Aug 10;27:347. doi: 10.1186/s13059-026-04234-4 (PMC13741493; doi:10.1186/s13059-026-04234-4)
Supplement: Supplementary file 2 [file 13059_2026_4234_MOESM2_ESM.docx]

**Supplementary Information**

**Mathematical derivation for approximating transcript proportions**

We first consider the expression of a single gene, *g*, in an arbitrary sample. Denote the observed total expression count as $Y_{g}$, the average expression from a tumor cell as $T_{g}^{c}$, the average expression from a non-tumor cell as $N_{g}^{c}$, and the cell counts of tumor and non-tumor cells in the sample respectively as $C_{T}$ and $C_{N}$. We can write $Y_{g}=T_{g}+N_{g}=C_{T} T_{g}^{c}+C_{N} N_{g}^{c}$. Therefore, the total expression count for the sample can be expressed as:

$$Y= \sum_{g} Y_{g}=\sum_{g} (T_{g}+N_{g})=\sum_{g} C_{T}T_{g}^{c}+ \sum_{g} {C_{N}N}_{g}^{c}$$

The true tumor expression proportion that we want to estimate is $\pi=\sum{C_{T}T}_{g}^{c}/Y$, with *g* omitted under the summation. Hence, for each gene *g,* we can rewrite the relationship as:

$$\frac{Y_{g}}{Y} = \frac{{C_{T}T}_{g}^{c}}{Y}+ \frac{{C_{N}N}_{g}^{c}}{Y}$$

$$Y_{g}^{'}= \frac{\sum C_{T}T_{g}^{c}}{Y} \frac{T_{g}}{\sum C_{T}T_{g}^{c}}+ \frac{\sum{C_{N}N}_{g}^{c}}{Y} \frac{N_{g}}{\sum{C_{N}N}_{g}^{c}}$$

$$Y_{g}^{'}=\pi\frac{T_{g}}{\sum T_{g}}+(1-\pi)\frac{N_{g}}{\sum N_{g}}$$

$$\begin{aligned} Y_{g}^{'}=\pi T_{g}^{'}+\left( 1-\pi\right)N_{g}^{'}\#\left( 1 \right) \end{aligned}$$

$Y_{g}^{'}$*,* $T_{g}^{'}$ and $N_{g}^{'}$ are the normalized expression values for an arbitrary gene *g*. As such, the model used by DeMixT^1,2^ can provide the exact transcript proportion when the hidden components are correctly estimated to follow the normalized expressions. The DeMixNB model introduces another layer of approximation by assuming eq (1) holds only at the mean level.

**Algorithm 1. A pseudocode description of the DeMixNB algorithm.**

**Reference**

1.	Cao, S., Wang, J. R., Ji, S., Yang, P., Dai, Y., Guo, S., *et al.* Estimation of tumor cell total mRNA expression in 15 cancer types predicts disease progression. *Nat. Biotechnol.* **40**, 1624–1633 (2022).

2.	Wang, Z., Cao, S., Morris, J. S., Ahn, J., Liu, R., Tyekucheva, S., *et al.* Transcriptome Deconvolution of Heterogeneous Tumor Samples with Immune Infiltration. *iScience* 451–460 (2018) doi:10.1016/j.isci.2018.10.028.
